# A registered report testing the effect of sleep on Deese-Roediger-McDermott false memory: greater lure and veridical recall but fewer intrusions after sleep

**DOI:** 10.1098/rsos.220595

**Published:** 2023-12-06

**Authors:** Matthew H. C. Mak, Alice O'Hagan, Aidan J. Horner, M. Gareth Gaskell

**Affiliations:** Department of Psychology, University of York, Heslington, York YO10 5DD, UK

**Keywords:** sleep, false memory, Deese-Roediger-McDermott, recall, gist abstraction, spreading activation

## Abstract

Human memory is known to be supported by sleep. However, less is known about the effect of sleep on false memory, where people incorrectly remember events that never occurred. In the laboratory, false memories are often induced via the Deese-Roediger-McDermott (DRM) paradigm where participants are presented with wordlists comprising semantically related words such as *nurse*, *hospital* and *sick* (studied words). Subsequently, participants are likely to falsely remember that a related lure word such as *doctor* was presented. Multiple studies have examined whether these false memories are influenced by sleep, with contradictory results. A recent meta-analysis suggests that sleep may increase DRM false memory when short lists are used. We tested this in a registered report (*N* = 488) with a 2 (Interval: Immediate versus 12 h delay) × 2 (Test Time: 9:00 versus 21:00) between-participant DRM experiment, using short DRM lists (*N* = 8 words/list) and free recall as the memory test. We found an unexpected time-of-day effect such that completing free recall in the evening led to more intrusions (neither studied nor lure words). Above and beyond this time-of-day effect, the Sleep participants produced fewer intrusions than their Wake counterparts. When this was statistically controlled for, the Sleep participants falsely produced more critical lures. They also correctly recalled more studied words (regardless of intrusions). Exploratory analysis showed that these findings cannot be attributed to differences in output bias, as indexed by the number of total responses. Our overall results cannot be fully captured by existing sleep-specific theories of false memory, but help to define the role of sleep in two more general theories (Fuzzy-Trace and Activation/Monitoring theories) and suggest that sleep may benefit gist abstraction/spreading activation on one hand and memory suppression/source monitoring on the other.

## 1. Introduction

Newly acquired episodic memory is usually better remembered after a period of sleep than after an equivalent period of wakefulness. For instance, one of the most robust findings in the memory literature is that word pairs encoded before sleep (versus wakefulness) are usually recalled with greater accuracy (e.g. [1–7]). Some theories attribute this benefit to sleep-related consolidation, during which the newly acquired memory may be reactivated via hippocampal replay, facilitating its integration into long-term neocortical stores (e.g. [8–14]). Alternatively, sleep may protect newly encoded memories from external interference, resulting in less forgetting [15,16].

Over the last 20 years, a growing body of evidence suggests that sleep may play a broader role in human memory than just consolidating or protecting previously encoded materials (e.g. [9,17,18]). One strand of research in this area is how sleep influences false memory, in which people remember events or items that never occurred (e.g. [19–21]). The Deese-Roediger-McDermott (DRM) paradigm is perhaps the most widely used paradigm for eliciting false memories in the laboratory [22,23]. Here, participants study lists of related words (‘studied words', e.g. *nurse, hospital, sick*). Not presented, however, is a ‘critical lure', which represents the gist of each list (e.g. *doctor*). In a subsequent memory test, participants are likely to erroneously recall the critical lures or identify the lures as previously seen, despite not having been exposed to them. This DRM false memory effect has been widely studied and replicated across age groups (e.g. [24,25]), speakers of different languages (e.g. [26,27]), presentation modalities (e.g. [28,29]), and various delay intervals between wordlist presentation and the subsequent memory test (e.g. from a few minutes to 60 days later; [30,31]). Of these, several studies have tested whether a delay interval containing a period of sleep (versus wakefulness) influences the incidence of false memory in the DRM paradigm (e.g. [32–34]). However, these studies have produced conflicting results, and the role of sleep remains elusive. Before going into the details of the inconsistencies in the existing ‘Sleep × DRM' literature, we first consider how DRM false memory arises and how sleep may influence this process.

### 1.1. How does DRM false memory arise?

Two theoretical accounts dominate the DRM literature: Fuzzy-Trace Theory (FTT) [35] and Activation/Monitoring Framework [36]. Below, we briefly consider how each of them accounts for the emergence of DRM false memory (for a comprehensive review, see [37]).

According to FTT, encoding a DRM wordlist creates two types of memory representations: (i) a verbatim trace that captures the surface forms of the experienced items (e.g. font, colour, voice) and (ii) a gist trace that captures the items' semantic content and their relationships. These traces are stored in parallel, but forgetting rates are generally higher for verbatim than for gist traces. At the subsequent memory test, an individual can retrieve the verbatim and/or the gist traces, depending on factors such as their availability and contextual cues. Verbatim retrieval leads to a vivid recollection, supporting recall and recognition of the studied list words and simultaneously suppressing false memories (i.e. the unpresented critical lures). On the other hand, while the retrieval of the gist traces also supports veridical recall, it may trigger DRM false memory because list words (e.g. *nurse*, *hospital*) and the lures (e.g. *doctor*) share overlapping semantic content.

The Activation/Monitoring Framework is also a dual-process theory but the two processes are cognitive operations, instead of memory representations. The first process, *activation*, is built upon the notion of spreading activation in associative networks, where words are interconnected based on semantic relatedness [38–40]. It posits that when a DRM wordlist is encoded (or retrieved), activation from these list words will spread to the unpresented critical lures since they were semantically related, potentially resulting in DRM false memories. However, at the point of retrieval, activation of the lures could be suppressed by the second process, known as *monitoring*. It assumes that since many words were previously activated, an individual will need to use source monitoring [41] to separate out items that lack diagnostic features of prior presentations, such as the sensory details or cognitive processes at encoding. While monitoring can suppress false memories, it can also suppress veridical memories when the studied list words lack such diagnostic features.

Both theories can account for nearly all findings in the existing DRM literature (see [42] for a review); less clear, though, is how they may explain a potential role of sleep in the emergence of DRM false memory. Below, we first consider both theories before turning to other theories that are primarily concerned with the effect of sleep on memory consolidation.

### 1.2. Sleep and DRM false memory

DRM false memories can emerge at any point of memory formation: encoding, consolidation, and retrieval [43]. If sleep has an effect on DRM false memories, it is likely to reside in the consolidation stage where the memory traces are stabilized and strengthened. Consolidation occurs in both waking and sleep, but sleep may be particularly conducive to consolidation due to (i) its unique physiological and neurochemical properties, and/or (ii) the fact that there is limited incoming sensory information during sleep. Our study is not intended to tease these apart and any potential sleep-related effects can be attributed to either or both (interested readers can refer to Dastgheib *et al*. [44] and Paller *et al*. [12] for in-depth discussions).

We begin by considering how FTT may predict an effect of sleep on DRM false memory. At present, it is difficult to generate a clear prediction from it, because there are multiple possibilities: sleep-related consolidation, relative to wakefulness, may boost (1) both the verbatim and gist traces equally (2) both traces but with varied strengths (3) selectively the verbatim traces, or (4) selectively the gist traces. Each of these possibilities leads to a different behavioural prediction. For instance, if sleep selectively boosts the verbatim trace, it should increase vivid recollection and memories for the list words, which may, in turn, help suppress the gist trace, reducing the instance of DRM false memory. On the other hand, if sleep selectively boosts the gist trace, it may lead to an increase in DRM false memories. In short, how FTT may predict the effect of sleep on DRM false memory remains an open question. By contrast, the Activation/Monitoring Framework seems to make a clearer prediction such that DRM false memory may increase after sleep (versus wake; [45,46]). Some existing evidence suggests that a period of sleep (versus wakefulness) may be more conducive to spreading activation [47,48], potentially because incoming sensory information is limited [45] and/or because a particular sleep stage plays a key role in promoting spreading activation ([47,49]; but see [50]). If spreading activation is more effective during sleep (versus wake), this should increase the chance of activation circulating into or being maintained within the critical lures, resulting in more DRM false memories.

Similarly, the information Overlap to Abstract (iOtA) model also makes the same behavioural prediction. Specifically, it proposes that sleep selectively strengthens the overlapping element of a set of related memories [9], which in the case of a DRM wordlist would be the critical lure. The iOtA model, therefore, makes an explicit prediction that post-encoding sleep (versus wakefulness) will increase the likelihood of the lures being falsely remembered. While the iOtA and Activation/Monitoring models may make the same behavioural prediction, their underlying cognitive mechanisms are somewhat different. For iOtA, it is posited that memory is reactivated during sleep-related consolidation such that elements unique to each memory (e.g. list words) and elements that are shared (e.g. critical lures) would be reactivated. These shared elements are hypothesized to be *selectively* strengthened over sleep. As for the Activation/Monitoring Framework, it may assume that sleep increases spreading activation to not only the lures but also the *non-shared* elements, albeit to a lesser extent (e.g. *gentle* is not a medical word, but it may be falsely remembered because it is associated with *nurse*). In sum, the iOtA and Activation/Monitoring models arrive at the same behavioural prediction via different cognitive mechanisms, with the former focusing solely on the *shared* elements and the latter less so.

Interestingly, however, a post-sleep *reduction* in DRM false memory has also been predicted. If sleep soon follows DRM encoding, the verbatim trace and/or activation for the list words may be stabilized and strengthened by sleep-related consolidation, enhancing retention of the studied list words (e.g. [34,51]). In turn, this may help suppress the gist-trace/lure activation, leading to fewer DRM false memories at retrieval. Lo *et al*. [3] argued that this view is in line with the synaptic homeostasis hypothesis [52,53], which posits that peripheral aspects of an encoded memory, such as the sensory details of a studied list word [54,55], would be pruned during sleep. Lo *et al*. [3] further hypothesized that pruning of the contextual details will improve accessibility for the list words, subsequently aiding the suppression of the critical lures at test. In contrast to the synaptic homeostasis hypothesis, Fenn *et al*. [32] appealed to a standard active consolidation theory and proposed that the sensory details associated with a list word, instead of being pruned, may be better consolidated (or preserved) over sleep (versus wakefulness). In turn, these item-specific sensory details would make the list words more distinctive from the lures, facilitating the recall of the verbatim trace and/or source monitoring. This may then aid the suppression of false memories at test. Currently, it is unclear whether sleep prunes or boosts sensory details associated with list words, and this would require more than a behavioural study to tease apart (e.g. [56]). Regardless, these theories predict that post-encoding sleep (versus wakefulness) will lead to a reduction in DRM false memories, potentially via an increase in veridical memories that will, in turn, boost a participant's ability to suppress the lures.

Finally, while it is possible to generate from some theories a predicted direction of effect, other theories are oftentimes not well-specified enough to make this possible. One example is Yonelinas *et al*.'s [16] contextual binding theory, which attributes sleep-related benefits in declarative memories to reduced forgetting of an item's contextual information. At present, their definition of ‘contextual information' was very extensive, encompassing any ‘aspect of the study episode…that links the test item to the specific study event' (p. 1). This means that it is possible to conceive ‘contextual information' as the sensory details of the studied words and/or as the gist trace of a list (see [57]). This underspecification makes it difficult to derive from the theory a clear behavioural prediction in the DRM paradigm, highlighting a need for further tightening.

Now, having considered the disparate behavioural predictions from established theories regarding the effect of sleep on DRM false memory, we turn to the studied list words. The theories outlined above differ somewhat in terms of their prediction. On one hand, the synaptic homeostasis hypothesis may suggest that signal-to-noise ratio for the list words would be improved after sleep, thereby boosting veridical memory (e.g. [3]). Similarly, the contextual binding theory also predicts that sleep (versus wake) will benefit the retention of list words as it may reduce forgetting of the list words' contextual information. On the other hand, the iOtA framework takes a more agnostic view. It argues that sleep *selectively* strengthens the shared elements of related memories; less clear is how sleep might simultaneously influence the non-overlapping elements themselves (i.e. the list words). As for the Activation/Monitoring Framework, different predictions are possible, depending on what assumptions are in place. First, if we assume that sleep (versus wake) increases spreading activation by increasing the amount of activation available in an associative network, it would be reasonable to predict that studied list words would receive more activation and hence be better remembered post-sleep. However, if sleep simply makes existing activation spread more widely, activations would become more diffuse, resulting in the studied list words being less activated (see [58]). This may potentially lead to poorer veridical memories post-sleep or at least at the same level as post-wake. In short, the Activation/Monitoring Framework is perhaps underspecified regarding how sleep may simultaneously affect veridical and false memory in the DRM paradigm.

The question of whether sleep (versus wake) affects *both* DRM false and veridical memory parallels the literature on regularity extraction and generalization. Some have argued that generalization, like gist extraction, is selectively facilitated by sleep consolidation ([59], but see also [60,61]). However, retrieval-based models of generalization would show sleep benefits on generalization only if sleep also strengthened the individual memories on which the generalization operated [62,63]. Given these theoretical discrepancies, a feature of our planned experiment was to assess the effect of sleep versus wake on *both* studied words *and* critical lures, although our focus is on the latter given the critical lures have been the primary focus of previous DRM studies reporting sleep effects.

### 1.3. Empirical evidence regarding the effect of sleep in DRM false memory

To the best of our knowledge, there are about 10 published DRM studies to date that have compared the effects of overnight sleep versus daytime wakefulness. Some of them demonstrated an increase in false memory after sleep [33,34,64], consistent with the iOtA and Activation/Monitoring models. However, other studies reported no overall effect [65] and some a reduction in DRM false memories following sleep (e.g. [3,32]). Complicating the picture further, a few DRM studies suggest that the effect of sleep may be affected by a participant's level of memory performance (indexed by the number of correctly recalled list words minus intrusion; [65])^1^, and a participant's age [66]. Turning to veridical memories (i.e. studied list words), the evidence is also somewhat inconsistent. Some reported greater veridical memories after sleep ([33,34]; Experiment 1) while others reported null results (e.g. [33]; Experiment 3). In sum, despite prior efforts in examining the effect of sleep in the DRM paradigm, the evidence base is weak and somewhat contradictory. This highlights a clear need for research aimed at reconciling the existing literature.

Motivated by the inconsistent evidence, Newbury and Monaghan [46] conducted a meta-analysis on 12 DRM experiments that compared the effects of sleep versus wakefulness (see [67] for a meta-analysis on fewer studies). They reported that while sleep did not have a consistent effect on either false or veridical memories, the effect of sleep was moderated by a key factor: the number of related words in a DRM list. Specifically, they found a consistent post-sleep increase in DRM false memories when a study used short wordlists (*N* = 10 words/list), but no consistent sleep effect among studies that used longer lists (*N* = 12 or 15 words/list). To explain this finding, Newbury and Monaghan [46] appealed to the level of initial encoding. First of all, wordlists containing fewer related words are known to reduce the incidence of DRM false memory [68]. Potentially, this is because the gist trace is less prominent. Or, since a short list necessarily activates few list words, the amount of activation in an associative network should be relatively low, in turn reducing the likelihood of activation spreading to the lures. In other words, false memory for the lures may be fairly weak at study, leaving more room for post-encoding sleep to exert an influence, which as described, may promote gist extraction [9] and/or spreading activation [47,48]. The possibility that a sleep effect may be more consistent when the lures are weakly encoded/activated at study is compatible with prior findings that declarative memories encoded with a lower strength (but not at floor) are more likely to benefit from sleep-related consolidation, as these memories may be prioritized for consolidation due to them having a greater need of being stabilized (e.g. [5,69,70]).

If sleep indeed increases DRM false memories in short lists, this will pose a challenge to theories that predict a post-sleep reduction in DRM false memories (e.g. [32]); by contrast, this will provide support for theories that predict the opposite (e.g. iOtA, spreading activation). Therefore, examining the effect of sleep in short wordlists provides us with an opportunity to evaluate competing theories. Furthermore, although Newbury and Monaghan's [46] meta-analysis provided evidence that can potentially reconcile the existing evidence base and pointed us towards the most conducive parameter for detecting a sleep effect in the DRM paradigm (i.e. short list length), the literature lacks a well-powered empirical study that tests the validity of this parameter. This is especially important when most prior ‘Sleep × DRM' studies had relatively small sample sizes. For instance, across nine studies (representing 12 separate experiments) included in Newbury and Monaghan's [46] meta-analysis, the median number of participants/group was 27.6 (*SD* = 17.5). Small sample sizes *per se* are not an issue if the comparison of interest has a large effect size; however, this is unlikely to be the case for the effect of sleep in DRM false memory, which seems to have an effect size smaller than Cohen's *d* = 0.5 (at least when longer lists are used; [46]). It is estimated that in order to achieve >80% statistical power (alpha = 0.05) to detect such an effect size, at least 65 participants per group are needed [71]. We, therefore, have reasons to hold a slightly sceptical view of prior findings, which leads us to propose a well-powered DRM experiment to evaluate the effect of sleep in short DRM lists. By doing so, we will be able to build a more sound empirical base for existing and future theories to exploit.

## 2. Experiment

### 2.1. Overview

It is possible to index DRM false memory via free recall or recognition (e.g. [72]). In this experiment, we used free recall only, because recall tends to be more prone to sleep-related memory effects than recognition ([46]; see also [73–75]). Our experiment comprised a study and a test phase. In the study phase, a participant encoded 20 short DRM wordlists, with each containing eight words. Short lists were chosen because Newbury and Monaghan's [46] meta-analysis pinpointed a clear sleep effect in these lists. In the test phase, participants recalled the wordlists in a free recall procedure.

Participants were randomly assigned to one of the four groups: AM-control, PM-control, Sleep or Wake. Those assigned to the control (aka Immediate) groups carried out the test phase immediately after the study phase, with those in the AM group starting at 9:00 (±1 h) and those in the PM group starting at 21:00 (±1 h). No difference in false or veridical recall was expected between these groups, as prior DRM studies (e.g. [32,33,76]) have consistently demonstrated that immediate recall was equivalent between morning and evening. The inclusion of these control groups helped rule out potential circadian effects on encoding and retrieval (and relatedly, monitoring in the Activation/Monitoring Framework). Finally, participants assigned to the Sleep and Wake groups (collectively referred to as the Delay groups) started the test phase approximately 12 h after the study phase. Those in the Wake group studied the DRM wordlists in the morning (9:00 ± 1 h) and completed the test phase in the evening (21:00: ± 1 h) on the same day. Those in the Sleep group encoded the wordlists in the evening (21:00 ± 1 h) and completed the test phase in the morning (9:00 ± 1 h) the next day.

### 2.2. Research questions and corresponding predictions

This experiment set out to address a key question (see appendix A for details):

Does overnight sleep (versus daytime wakefulness) influence DRM false recall?

Our prediction was based on the meta-analysis by Newbury and Monaghan [46], who reported that when a study used short lists, sleep consistently increased DRM false memory. We, therefore, predicted a post-sleep (versus post-wake) increase in DRM false recall, whereas there would be no such difference between the AM- and PM-control groups.

Our study also addressed a peripheral question:

Does overnight sleep (versus daytime wakefulness) increase veridical recall of the studied list words?

Again, our prediction was based on Newbury & Monaghan [46], who found that sleep benefits veridical memory in short lists. We therefore predict that veridical recall would be greater post-sleep than post-wake, whereas there would be no such difference between the AM- and PM-control groups.

### 2.3. Design

#### 2.3.1. Does overnight sleep (versus daytime wakefulness) influence DRM false recall?

For this question, the dependent variable was whether a critical lure is recalled or not (i.e. binary). There were two independent variables: Interval (Immediate versus Delay) and Test Time (9:00 versus 21:00)^2^, both of which were manipulated between-participants. In other words, the four groups were coded as in table 1:

**Table 1.** How the four groups were coded using Interval and Test Time.

groups		Interval		Test Time
AM-control	=	immediate	+	9:00
PM-control	=	immediate	+	21:00
sleep	=	delay	+	9:00
wake	=	delay	+	21:00

To address Research Question #1, we first tested if any difference between the Sleep and Wake groups was significantly different from that between the AM- and PM-control groups (i.e. an interaction between Interval and Test Time). This is important because it allows us to rule out time-of-day effects. Then, we tested for the simple effect of Test Time (9:00 versus 21:00) within the Immediate and Delay groups. If there is (1) a significant Interval × Test Time interaction and (2) a significant Test Time effect within the Delay groups (Sleep > Wake), we will be able conclude that sleep (but not time-of-day) increases false recall.^3^

#### 2.3.2. Does overnight sleep (versus daytime wakefulness) increase veridical recall of the studied list words?

For this research question, the dependent variable was whether a studied list word was recalled or not (i.e. binary). As per Question #1, there were two between-participant manipulations: Interval (Immediate versus Delay) and Test Time (9:00 versus 21:00). We first tested if there was an interaction between Interval and Test Time. Then, we tested for the simple effect of Test Time within the Immediate and Delay groups. Note that this research question is secondary to the first.

### 2.4. Target sample size and stopping rules

Our target sample size was 120 participants/group (i.e. 480 participants in total), defined as those who remained in the sample after applying the exclusion criteria outlined in §2.9. This sample size gives us ≥90% power to detect all the desired effects for our Research Questions (see appendix B for a detailed power analysis).

### 2.5. Recruitment

#### 2.5.1. Online recruitment

Participants were recruited online via Prolific (https://www.prolific.co/). All participants completed the experiment unsupervised and at a location of their own choosing. We chose online testing, as opposed to lab-based testing, for at least two reasons. First, given the unpredictability of the COVID-19 pandemic, we did not want to risk the possibility of data collection being disrupted. Second, given the time limit on the funding for this work, it would have been logistically difficult to reach the target sample size were the study conducted in person.

One key concern associated with online testing is data quality. This stems from the fact that researchers cannot monitor participants during an online experiment. However, it has been repeatedly demonstrated that as long as appropriate measures are taken (e.g. [79,80]), data quality from online experiments is no different from lab-based experiments (e.g. [81–84]). Furthermore, two recent online studies using the same experimental design [1,4] found clear evidence of a sleep benefit in the classic paired-associate learning paradigm, replicating well-established evidence from laboratory-based experiments (e.g. [3,6]). Importantly, the effect sizes for sleep from these online studies were roughly equivalent to those reported by laboratory-based studies. Together, these suggest that it is possible to detect sleep-related memory effects in online experiments, as long as the appropriate measures are put in place. These are detailed in the ***Procedures*** section (#2.8) below.

#### 2.5.2 Recruitment method

Following two previous sleep studies conducted via Prolific [4,85], we put a short survey on the platform to recruit a pool of participants (*N* = 2296). This survey is available in appendix C and was hosted on Qualtrics. The first half of the survey asked for basic demographic information: gender identity, age, current country of residence, first language, ethnicity, highest education attainment and history of developmental/sleep disorders (if any). The survey then provided a brief outline of the main study. It stated that if enrolled, participants would be randomly allocated to one of the four groups and that no preferences would be accommodated. Participants then indicated whether they would like to enroll in the main study. Of the 2296 respondents, 1940 expressed interest in taking part, who were then screened for their eligibility (see inclusion criteria below). Those who fitted our inclusion criteria were then randomly allocated to one of the four experimental groups. A private message was sent to each participant, notifying them of their group allocation. In the end, 534 participants completed both the study and test phases. These participants were reimbursed at a rate of approximately £9.5/h.

### 2.6. Inclusion criteria

We applied these inclusion criteria to ensure comparability with prior studies (e.g. [32–34,64]):

1. Aged 18–25
2. Speaks English as (one of) their first language(s)
3. No known history of any psychiatric (e.g. schizophrenia), developmental (e.g. dyslexia) or sleep (e.g. insomnia) disorders
4. Currently resides in the UK, indexed by their IP address (since this experiment requires participants to complete each phase at a certain time of day, it is necessary to restrict the location to prevent participants from taking the study in different time zones)
5. Normal vision or corrected-to-normal vision
6. Normal hearing
7. Able to complete the study using a laptop or a desktop PC
8. Able to complete both the study and test phases
9. Has an approval rate of >96% on Prolific. This helps ensure that a participant has a tendency to take online studies seriously.

### 2.7. Materials

Prior studies in the DRM literature typically showed 8–15 words per list (e.g. [32,64,86]). Generally, within this range, showing fewer words reduces false recall rates ([68,86]; see also [87]). However, showing even fewer words per list (e.g. 3) results in floor or near-floor rates [68]. Given that sleep seems to have a larger effect on false memory when the gist trace or lure is encoded at a medium level during study ([46]), we opted for eight words per list.

We made use of 20 DRM wordlists (table 2), taken from Roediger *et al*. [36]. Each list contained eight semantically related words, and as per the standard DRM paradigm, they were arranged in a descending order of associative strength to the critical lures. A participant studied all 20 lists. We note that the original DRM lists by Roediger *et al*. [36] were tailored for American participants, and two words (e.g. *trash, Mississippi*) were not immediately relatable to people in the UK. We, therefore, changed these words (e.g. *trash* → *rubbish*), as noted in table 2.

**Table 2.** The 20 DRM wordlists used in the experiment. **Note 1.** In Roediger *et al*. [36], the critical lure for this list was *trash,* with *rubbish* being one of the list items*.* We used *rubbish* as the critical lure and *trash* as a list item because the former is the preferred term in British English. **Note 2.** The original word in Roediger *et al*. was *Mississippi*. We replaced it with *Thames*.

critical lure of each list	false recall probability [36]	list items (arranged in the order of presentation in study)
*window*	*65*	*door, glass, pane, shade, ledge, sill, house, open*
*sleep*	*61*	*bed, rest, awake, tired, dream, wake, snooze, blanket*
*doctor*	*60*	*nurse, sick, lawyer, medicine, health, hospital, dentist, physician*
*smell*	*60*	*nose, breathe, sniff, aroma, hear, see, nostril, whiff*
*chair*	*54*	*table, sit, legs, seat, couch, desk, recliner, sofa*
*smoke*	*54*	*cigarette, puff, blaze, billows, pollution, ashes, cigar, chimney*
*sweet*	*54*	*sour, candy, sugar, bitter, good, taste, tooth, nice*
*rough*	*53*	*smooth, bumpy, road, tough, sandpaper, jagged, ready, coarse*
*needle*	*52*	*thread, pin, eye, sewing, sharp, point, prick, thimble*
*rubbish*	*49*	*garbage, waste, can, refuse, sewage, bag, junk, trash* (**Note 1**)
*anger*	*49*	*mad, fear, hate, rage, temper, fury, ire, wrath*
*soft*	*46*	*hard, light, pillow, plush, loud, cotton, fur, touch*
*city*	*46*	*town, crowded, state, capital, streets, subway, country, New York*
*cup*	*45*	*mug, saucer, tea, measuring, coaster, lid, handle, coffee*
*cold*	*44*	*hot, snow, warm, winter, ice, wet, frigid, chilly*
*mountain*	*42*	*hill, valley, climb, summit, top, molehill, peak, plain*
*slow*	*42*	*fast, lethargic, stop, listless, snail, cautious, delay, traffic*
*river*	*42*	*water, stream, lake, Thames* (Note 2)*, boat, tide, swim, flow*
*spider*	*37*	*web, insect, bug, fright, fly, arachnid, crawl, tarantula*
*foot*	*35*	*shoe, hand, toe, kick, sandals, soccer, yard, walk*

We acknowledge that previous studies in the ‘Sleep × DRM' literature typically showed participants 8 to 16 lists (e.g. [33,34]), so our participants studied more wordlists (i.e. 20). However, since we showed relatively few words per list, the total number of studied words was comparable to prior studies (i.e. 160 in the current versus 96 to 225 in prior studies). Furthermore, an advantage of showing more wordlists is that more critical lures could be recalled (i.e. 20 lists = 20 lures), potentially increasing variability between participants and hence our ability to detect sleep-related effects.

### 2.8. Procedures

**Figure 1.**
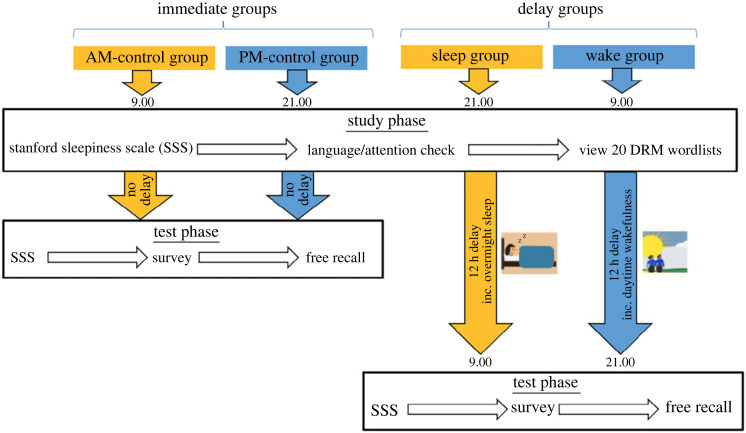
Experimental procedure.

The procedure of the study is summarized in figure 1. The study was hosted on Gorilla (www.gorilla.sc; [81]). A study phase took approximately 11 min. Here, participants first gave informed consent, completed a language/attention check, rated their level of sleepiness on the Stanford Sleepiness Scale (SSS; [88]), and viewed 20 DRM wordlists.

Immediately afterwards, participants in the AM/PM-control groups carried out the test phase. For those in the Delay groups, the test phase took place approximately 12 h later. Here, both the Immediate and Delay participants rated their level of sleepiness on SSS and completed a short survey concerned with, for example, morningness/eveningness preference (rMEQ; [89]) and sleep duration/quality the night before (see Appendix D for the full survey). This survey helped determine whether the four groups were matched in terms of time-of-day preference and whether data from a participant needed to be discarded as a result of meeting the exclusion criteria described in §2.9. Finally, the test phase concluded with a 10 min free recall task where participants recalled as many of the words as they could from the previously seen wordlists.

#### 2.8.1. Exposure to the DRM wordlists

On the instruction page, participants were told that they would see some English words presented one after the other on the computer screen. They were asked to pay close attention to the words because they would be tested on them later on. No specific instruction was given regarding the subsequent test format.

During presentation, words in each DRM list were presented visually,^4^ in a fixed order and arranged in descending associative strength to the unpresented critical lure (table 2 for order). Each list began with a fixation for 1 s, followed by the first word in a list. Each word was shown for 1 s, in a lowercase black font (Arial, size 26) on a white background, and separated by a 500 ms interstimulus interval. After presentation of the final word in a list was 5 s of blank screen. List order was randomized, and each list was seen once.

There was a surprise attention check after the 4th, 9th, 13th, 18th lists, where participants saw an erroneous maths equation such as ‘3 + 3 = 11'. It was presented for 1 s, in the same font and style as the list words [90]. Immediately afterwards, participants were asked to report what 3 + 3 was according to what was just shown.

#### 2.8.2. Free recall

Participants had 10 mins to type out all the words they could remember from the study phase in a textbox. When there was 2 min left, a timer appeared. Participants could not proceed before the time was up.

To maximize the likelihood that participants paid full attention instead of doing something else (e.g. playing with their phone) during recall, there was an attention check throughout: on the same page as the response textbox, there was a white square that turned red every 2–3 mins. The change in colour lasted for 10 s, during which a single digit was shown. Participants had to enter the digit into a separate textbox to show that they were paying attention. Throughout the 10 min recall task, the square turned red four times, so participants needed to enter four digits as they attempted the recall task.

#### 2.8.3. Additional measures to ensure data quality

At the start of the study phase, participants were encouraged to take the experiment seriously and were informed that their participation would contribute to science. After rating their level of sleepiness, participants must pass a language/attention check. This involved the auditory presentation of a short story. Replay and pausing were not permitted. Participants then answered two simple comprehension questions based on the story. Failure to answer both questions correctly led to their data being excluded from further analysis. These questions helped to ensure that participants could indeed understand English and were in a reasonably quiet environment. Next, to prevent participants from multitasking on the computer, both the study and test phases required participants to enter full-screen mode. Participants were told that exit from full-screen mode during the study may lead to no payment. This was made possible by Gorilla, which recorded the browser's and the monitor's sizes. At the end of the study phase, participants were asked to describe how they learnt the words in a sentence. Participants who said they wrote down or similarly recorded the words were excluded from further analysis.

### 2.9. Exclusion criteria

Exclusion was applied on the participant level. A participant's dataset was excluded from further analysis and replaced, if

1. they exited full-screen mode in any of the phases
2. they failed the language/attention check at the start of the study
3. they reported to have written down or recorded the wordlists during the study phase
4. (*Sleep and Wake groups only*) they reported consuming any alcoholic drinks between study and test
5. (*Sleep group only*) they reported to have had fewer than 6 h of overnight sleep prior to test or rated their sleep quality as poor or extremely poor
6. (*Wake group only*) they reported to have had a nap between study and test
7. they failed more than one of the four attention checks (i.e. 3 + 3 = 11) at study
8. they failed to report more than one of the four digits in the attention check of free recall
9. they submitted a blank response in free recall
10. (see Footnote^5^ for an explanation of why this criterion was not followed)
11. their completion time for either the study or test phase was 3 s.d. above or below their respective mean completion time of the first 480 participants who completed the study.

### 2.10. Participants

Of the 534 participants who completed both the study and test phases, 46 were excluded for meeting one or more of the exclusion criteria. The full list of excluded participants and their respective reason for exclusion is available on OSF (see exclusion_OSF.csv). Our final sample size comprised 488 participants, with 124 in each of the Immediate groups, and 120 in each of the Delay groups. Group characteristics are summarized in table 3.

**Table 3.** Group characteristics. *Notes.* (1) SSS stands for Stanford Sleepiness Scale and ranges from 1 to 6, with higher values indicating greater sleepiness. (2) rMEQ stands for reduced Morningness/Eveningness Questionnaire; it ranges from 5 to 25, with higher values indicating greater morningness preference.

characteristics	immediate- AM	immediate- PM	sleep (aka delay-AM)	wake (aka delay-PM)
*N* before exclusion	130	127	134	143
*N* after exclusion	124	124	120	120
mean age (s.d.)	22.24 (2.18)	22.34 (2.03)	22.18 (1.93)	22.25 (1.93)
gender (female: male: other)	64 : 54 : 2	77 : 46 : 1	62 : 57 : 1	58 : 61 : 1
% participants identified as ethnically white	78.2%	73.4%	81.7%	80%
mean SSS rating at study (s.d.)	2.58 (0.98)	2.64 (1.12)	2.66 (0.96)	2.58 (0.98)
mean SSS rating at test (s.d.)	2.73 (1.04)	2.95 (1.29)	2.63 (1.21)	2.67 (1.18)
mean rMEQ score (s.d.)	15.89 (1.67)	15.59 (1.91)	15.72 (1.83)	15.53 (1.99)
mean *N* of intervening hr between study and test (s.d.)	n.a.	n.a.	12.22 (0.74)	12.14 (0.81)

Prior to the confirmatory analyses, we first checked if the four groups were matched on their morningness/eveningness preference and degree of sleepiness at study/test (as indexed by the Stanford Sleepiness Scale). These are summarized in table 3. We compared the four groups on each of the measures using one-way ANOVAs, which showed no significant differences (SSS at study: *F* = 0.21, *p* = 0.888; SSS at test: *F* = 1.63, *p* = 0.183; rMEQ: *F* = 0.97, *p* = 0.408). We also compared the Sleep and Wake groups on the number of intervening hours between study and test using a between-participant *t*-test, which revealed no significant difference [*t*_236.16_ = 0.86, *p* = 0.389]. In sum, our four groups were well-matched on these potentially confounding factors.

### 2.11. Data pre-processing

The free recall data were pre-processed. The first step was to remove any duplicate responses. The second was to correct all obvious spelling and typing errors to the nearest English words, defined as Levenshtein distance ≤2 (e.g. *cigerette → *cigarette*).^6^ Responses with added or dropped inflectional suffixes (i.e. -*s*, -*ed*, -*ing*, adjectival -*er*) were corrected. Responses with derivational changes were considered as intrusions. For instance, one of the studied words is *pollution*; if a participant recalled *pollutions*, the plural suffix was dropped; however, if a participant recalled *pollutant*, this was considered as an intrusion.

### 2.12. Results of pre-registered analyses

Following prior studies in the ‘Sleep × DRM' literature, we adopted a frequentist approach for all our analyses. The alpha level was set at 0.05. All analyses were conducted in R [91], and all the graphs were created using the ‘ggplot2' [92] and ‘ggdist' [93] packages.

#### 2.12.1. Positive control

We checked if our paradigm consistently elicited the well-established DRM effect across participants. Given free recall, the chance level of a critical lure being produced is 0. We submitted the number of critical lures produced by all participants (range: 0–20) to a one-sample *t*-test, with the chance level being 0. It showed that participants were susceptible to false recall [*t*_487_ = 26.96, *p* < 0.001], providing evidence for the classic DRM false memory effect.

#### 2.12.2. Control analysis

**Figure 2.**
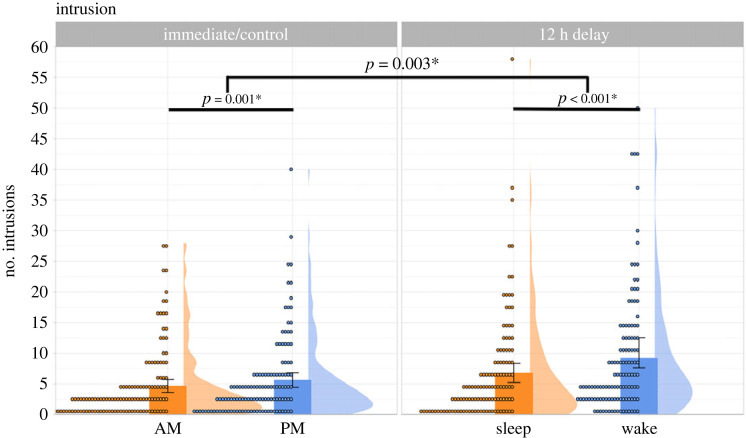
Mean number of intrusions produced, summarized across the four groups. Each dot represents an individual participant, and the error bars represent 95% confidence intervals.

Payne *et al*. [33] found that participants falsely recalled more critical lures post-sleep (versus post-wake). However, it is possible that participants simply had a greater tendency to put down more unseen words after sleep, not because sleep increases DRM false recall *per se*. Therefore, before addressing our key research questions, we checked if participants across groups were comparable in terms of their bias in producing unseen items. In Payne *et al*. [33], this bias was indexed via the number of intrusions (i.e. neither the studied nor the lure items), which was roughly equivalent between their Sleep and Wake groups (*M*_Sleep_ = 5.6 versus *M*_Wake_ = 6.2; *p* = 0.60) as well as between their AM and PM-control groups (*M*_AM_ = 4.1 versus *M*_PM_ = 4.1; *p* = 0.99). To check if this is the case in our data, we used a 2 (Interval: Immediate versus Delay) × 2 (test time: AM versus PM) Poisson regression. We chose Poisson regression, as opposed to ANOVA, because the intrusion data were count data, meaning that data distribution was right-skewed and hence unsuitable for ANOVA. Figure 2 summarizes the number of intrusions in each group.

A 2 × 2 Poisson regression revealed significant effects of Interval (*β* = 0.372, s.e. = 0.054, *z* = 6.845, *p* < 0.001) and Test Time (*β* = 0.185, s.e. = 0.056, *z* = 3.299, *p* < 0.001), which were qualified by a significant interaction (*β* = 0.214, s.e. = 0.072, *z* = 2.96, *p* = 0.003). Given this, we tested the simple effects of Test Time within the Immediate and Delay groups using the ‘emmeans' package [94]. Within the Immediate groups, the evening participants (M = 5.62, s.d. = 6.63) produced more intrusions than the morning participants (M = 4.67, s.d. = 5.97) (*z* = −3.299, *p* = 0.001). Likewise, in the Delay groups, the Wake participants, who completed free recall in the evening (M = 10.1, s.d. = 13.65), produced more intrusions than the Sleep participants, who completed recall in the morning (M = 6.78, s.d. = 8.71) (*z* = −8.808, *p* = < 0.001). Together, our data indicate that participants who attempted free recall in the evening (versus morning) were more prone to intrusions, and this effect was greater in the Delay than in the Immediate groups.

These unexpected findings prompted us to explore whether *the number of total responses* (i.e. studied + lures + intrusions) differed between morning and evening test time. Interestingly, this exploratory analysis (see §2.13.1) showed no effect of Test Time. Together, these suggest that attempting free recall in the evening led to a selective increase in intrusions, but not necessarily a global increase in output bias. Finally, given that Test Time had a significant effect on intrusions, we followed our pre-registered analysis plan by adding the number of intrusions as a numeric covariate in the 2 × 2 mixed-effects models below.

#### 2.12.3. Confirmatory analysis 1

This analysis addresses our key research question:

#1 Does overnight sleep (versus daytime wakefulness) influence DRM false recall?

**Figure 3.**
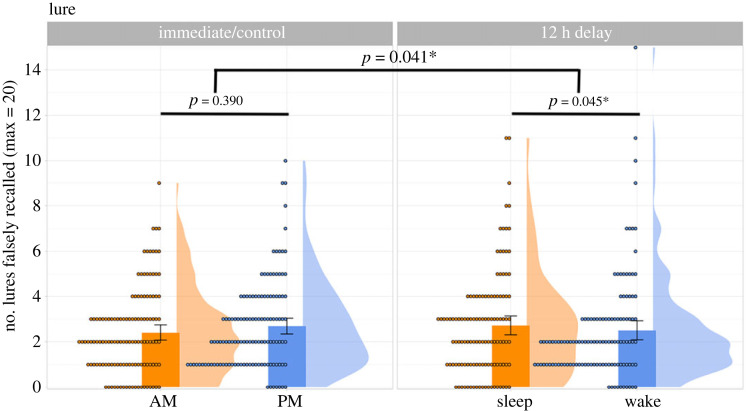
Mean number of critical lures falsely generated, summarized across the four groups. Each dot represents an individual participant, and the error bars represent 95% confidence intervals.

The number of critical lures falsely recalled is summarized across groups in figure 3.

A generalized linear mixed-effect model (GLMM) was fitted to the critical lure data on the item level (*N* of observations = 488 participants × 20 critical lures).^7^ The dependent variable was binary: whether a critical lure was recalled or not (1 versus 0). The fixed effects were the number of intrusions a participant produced, Interval (Immediate versus Delay), Test Time (AM versus PM), and an Interval by Test Time interaction. Interval and Test Time were coded using sum contrasts [95]. The random-effect structure was determined by the ‘buildmer' package [96], which automatically found the maximal model that was capable of converging using backward elimination (with the ‘bobyqa’ optimizer). This means that model selection started from the maximal model, as justified by the experimental design [97]. The model we reported and based our interpretation on was the most maximal model that was capable of converging (see the upper half of table 4 for the final random-effect structure and model output).

**Table 4.** Outputs from confirmatory GLMMs examining the effects of Intrusions, Interval and Test Time in false (upper) and veridical (lower) recall*.*

	*estimate*	s.e.	*z*	*p*-value
**false (lure) recall**				
random-effect structure: (intrusions | participant.ID) + (1 | lure)				
intercept	−2.328	0.110	−21.253	<0.001*
Intrusions	0.034	0.006	5.487	<0.001*
Interval (Immediate versus delay)	0.039	0.042	0.943	0.346
Test Time (AM versus PM)	0.035	0.041	0.850	0.395
Interval × Test Time	−0.084	0.041	−2.046	0.041*
**veridical (studied word) recall**				
random-effect structure: (intrusions | participant.ID) + (interval | studied.Item)				
intercept	−2.373	0.075	−31.705	<0.001*
intrusions	−0.007	0.005	−1.372	0.170
interval (immediate versus delay)	0.307	0.040	7.721	<0.001*
Test Time (AM versus PM)	0.026	0.037	0.691	0.489
Interval × Test Time	−0.124	0.037	−3.324	<0.001*

The number of intrusions had a significant effect on lure recall, such that participants who produced more intrusions tended to recall more critical lures (*z* = 5.487, *p* < 0.001). There were no main effects of Interval or Test Time (zs < 0.95, ps > 0.34), but there was a significant Interval by Test Time interaction (*z* = −2.046, *p* = 0.041). Following our pre-registered analysis plan, we proceeded to test the simple effects of Test Time within the Immediate and Delay groups, using the ‘emmeans' package [94] in R. Among the Immediate groups, there was no significant difference in lure recall between the AM-control and PM-control participants (*β* = −0.098, s.e. = 0.114, *z* = −0.859, *p* = 0.390). However, among the Delay groups, there was a significant difference (*β* = 0.239, s.e. = 0.119, *z* = 2.00, *p* = 0.045) such that the Sleep participants (M = 2.73, s.d. = 2.30) produced more critical lures than the Wake participants (M = 2.51, s.d. = 2.32).

Box 1. **R codes for confirmatory analysis 1.**

>contrasts(FalseRecall$Interval) < - contr.sum(2) #sum contrast for interval

>contrasts(FalseRecall$Test_Time) < - contr.sum(2) #sum contrast for test time

>library(lme4)

>library(buildmer)

># 2 × 2 GLMM

>FalseRecallModel < - buildmer(Recalled∼Interval * Test_Time + (1 | Participant) + (Interval * Test_Time | Item), data = FalseRecall, family = "binomial",buildmerControl = buildmerControl(direction = 'backward', args = list(control = glmerControl(optimizer = "bobyqa"))))

># Obtain the simple-effects of Test Test within the Immediate and Delay groups

>library(emmeans)

>emmeans(FalseRecallModel, pairwise∼Test_Time | Interval)

**Note.** Due to an oversight in our Stage-1 proposal, there was a discrepancy between the proposed R code and the verbal description of how the random-effect structures in our mixed-effect models would be simplified. In the verbal description, we wrote that the random-effect structures would be simplified using **backward** elimination in the R package ‘buildmer', while the R code prescribed **ordered** elimination. We tested both elimination methods on all our confirmatory models, and fortunately, they resulted in essentially the same model outputs, so we stuck with backward elimination throughout our analysis.

#### 2.12.4. Confirmatory analysis 2

This analysis addresses the secondary question:

#2 Does overnight sleep (versus daytime wakefulness) increase veridical recall of the studied list words?

**Figure 4.**
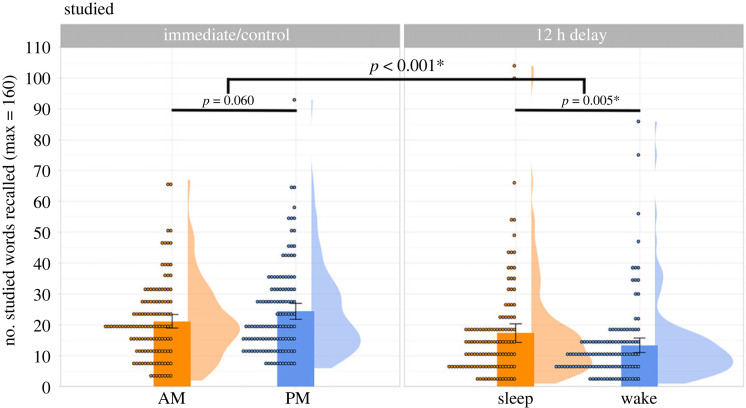
Mean number of studied list words correctly recalled, summarized across the four groups. Each dot represents an individual participant, and the error bars represent 95% confidence intervals.

Figure 4 summarizes the number of studied list words correctly recalled across groups.

We fitted a GLMM to the veridical recall dataset (*N* of observations = 488 participants × 160 studied words). The dependent variable was whether a studied word was recalled or not. The fixed effects were the number of intrusions a participant produced, Interval (Immediate versus Delay), Test Time (AM versus PM), and an Interval by Test Time interaction. The coding scheme and computation procedure were the same as in the previous analysis. The model output and its random-effect structure are available in the lower half of table 4. There were no significant effects of intrusions (*z* = −1.372, *p* = 0.170) or Test Time (*z* = −0.691, *p* = 0.489). However, there was a main effect of Interval (*z* = 7.721, *p* < 0.001) such that participants in the Delay groups recalled significantly fewer studied words (M = 15.37, s.d. = 14.97) than those in the Immediate groups (M = 22.77, s.d. = 13.59), indicating time-dependent memory decay. Importantly, there was a significant Interval by Test Time interaction (*z* = −3.324, *p* < 0.001), which we broke down with the ‘emmeans’ package as pre-registered. Within the Immediate groups, the evening participants (M = 24.36, s.d. = 14.60) recalled more studied words than the morning participants (M = 21.18, s.d. = 12.36), although this was not statistically significant (*β* = −0.196, s.e. = 0.104, *z* = −1.881, *p* = 0.060). Within the Delay groups, there was a main effect of Test Time (*β* = 0.299, s.e. = 0.107, *z* = 2.797, *p* = 0.005), such that the Sleep participants (M = 17.33, s.d. = 16.59) outperformed their Wake counterparts (M = 13.41, s.d. = 12.93). Together, these results support the well-established finding that sleep is beneficial to the retention of newly encoded declarative memories.

#### 2.12.5. Complementary Bayesian analysis

Although our inference was based on a frequentist approach, we pre-registered to use a Bayesian analysis to complement and test the strength of our results (e.g. [98]). Bayes Factors were computed for (1) the Interval by Test Time interaction in the false and veridical mixed-effect models above, and for the simple effects of Test Time within the (2) Immediate and (3) Delay groups. Following the procedures in Gilbert *et al*. [99], a Bayes Factor was computed using the Bayesian information criterion (BIC) approximation from two competing GLMMs. For instance, in computing the Bayes Factor for the Interval × Test Time interaction, two models were needed: An alternative model containing the full fixed-effects structure (Intrusions + Interval + Test Time + Interval:Test Time), and a null model lacking the interaction.^8^ To estimate the Bayes Factor, we used the formula *e*^ΔBIC^_10_^/2^, where ^ΔBIC^_10_ is the BIC for the null model minus the BIC for the alternative model [100–102]. This produces a Bayes Factor_10_, which was interpreted with reference to Lee & Wagenmakers' [103] heuristics. The current BIC approximation method has the advantage of being a straightforward solution for mixed-effects models; however, its usage remains controversial as it is known to favour the simpler model (i.e. the null hypothesis; [101,104,105]). Table 5 summarizes the Bayes Factors derived from our mixed-effects models.

**Table 5.** Bayes factors for the Interval × Test Time interactions and the simple effects of Test Time in the lure and veridical recall data*.*

effects	BF_10_
false (lure) recall	
Interval × Test Time	0.077
Test Time in immediate groups	0.00010
Test Time in delay groups	0.00193
veridical (studied word) recall	
Interval × Test Time	0.820
Test Time in immediate groups	0.00038
Test Time in delay groups	0.01763

Surprisingly, all the Bayes Factors, except for the Interval × Test Time interaction in the studied word model, were below 0.1. These, according to Lee & Wagenmakers [103], can be taken as strong evidence for the null hypotheses. In other words, there is a discrepancy between our frequentist and Bayesian analyses. We stress that this Bayesian analysis is complementary in nature and our primary test of significance remains the frequentist test, as pre-registered.

### 2.13. Results of exploratory analyses

In this section, we present the results of four exploratory analyses, which explored (1) the number of total responses (i.e. studied + lure + intrusions) across groups, (2) the effect of dropping intrusions as a covariate from the confirmatory models, (3) the likelihood with which a lure being produced is predicted by its corresponding list items being recalled, and (4) the semantic distance between intrusions and critical lures. Additional analyses (not reported here but available on the OSF) examined, (5) whether veridical recall exhibited the classic U-shaped serial position curve (e.g. [106]),^9^ (6) whether the effect of sleep on lure recall is modulated by veridical recall, as suggested by Diekelmann *et al*. [65].^10^

#### 2.13.1 Number of total responses

**Figure 5.**
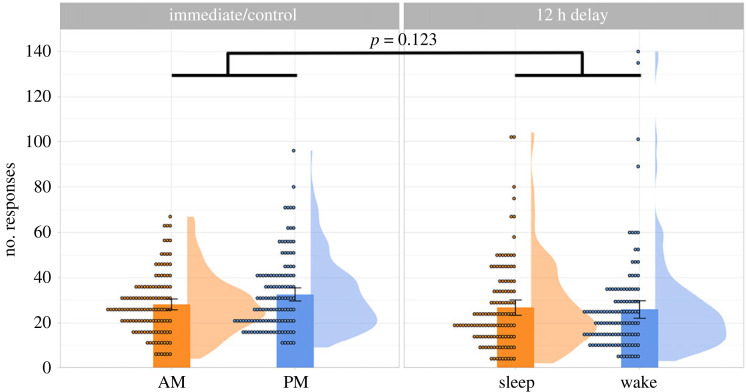
Mean number of total responses (i.e. studied + lure + intrusions), summarized across the four groups. Each dot represents an individual participant, and the error bars represent 95% confidence intervals.

In the light of the finding that participants who completed free recall in the evening (versus morning) produced more intrusions, we asked whether this was driven by these participants having a greater tendency to put down more responses generally. To test this, we calculated the number of total responses by each participant (i.e. studied + lures + intrusions), which is summarized across groups in figure 5.

Unlike the intrusion data which had an overall mean of 6.7 and a minimum of 0, the number of total responses had a mean of 28.5 and a minimum of 2, suggesting that it is better to consider total responses as continuous, as opposed to count, data. As such, we used a 2 × 2 between-participant ANOVA to test for the effects of Interval and Test Time on the number of total responses, which was log-transformed to give a more normal distribution. The ANOVA revealed a main effect of Interval [*F*_1, 484_ = 21.35, *p* < 0.001], such that participants in the Immediate groups (*M* = 30.5, s.d. = 15.0) gave more responses than those in the Delay groups (*M* = 26.4, s.d. = 20.1). However, importantly, there was no significant effect of Test Time [*F*_1, 484_ = 1.68, *p* = 0.196], and the Interval by Test Time interaction was also non-significant [*F*_1, 484_ = 2.39, *p* = 0.123]. Together with the intrusion data, this exploratory analysis suggests that completing free recall in the evening led to a selective increase in intrusions but not necessarily an increase in global response bias.

#### 2.13.2. Dropping intrusions from confirmatory models

As pre-registered, our confirmatory analyses included the number of intrusions as a covariate. Here, to better understand its influence on the overall results, we explored its removal from our confirmatory mixed-effect models.

For false recall, removing intrusions resulted in a non-significant Interval × Test Time interaction (*β* = −0.061, s.e. = 0.043, *z* = −1.41, *p* = 0.160). A follow-up emmeans comparison also indicated no significant Sleep-Wake difference (*β* = 0.134, s.e. = 0.128, *z* = 1.05, *p* = 0.293). This suggests that the absolute number of lures being generated did not significantly differ between the two groups. To further probe the role of intrusions in lure recall, we conducted an exploratory Mann-Whitney U test comparing the Sleep and Wake participants on their lure-to-intrusion ratios^11^; it showed that this ratio was significantly greater in the Sleep (Mdn = 0.61 : 1) than in the Wake (Mdn = 0.30 : 1) group (*z* = −2.82, *p* = 0.002). In other words, our confirmatory finding of greater lure recall in the Sleep (versus Wake) group is relative in nature and in part reflects a greater lure-to-intrusion ratio after sleep.

For veridical recall, even without considering intrusions, the Interval × Test Time interaction remained statistically significant (*β* = −0.125, s.e. = 0.037, *z* = −3.36, *p* < 0.001). A further pairwise comparison revealed a significant difference between the Sleep and Wake groups (*β* = 0.281, s.e. = 0.11, *z* = 2.55, *p* = 0.011), with the Sleep group outperforming the Wake group. This suggests that the effect of sleep on veridical recall did not depend on whether intrusions were taken into account.

#### 2.13.3. Dependency between lure and veridical recall on a list level

Here, we asked whether recall probability of a lure (e.g. *doctor*) is predicted by the number of corresponding list items being recalled (e.g. *nurse*, *hospital, sick*), and if it does, whether it differs between the Sleep and Wake groups. These questions may help shed light on the degree to which sleep increases lure recall via processes such as retrieval-induced generalization^12^ or gist abstraction,^13^ as these processes may predict a different degree of interdependence between lure and veridical recall. If sleep (versus wake) promotes retrieval-induced generalization, lure and veridical recall should become more strongly correlated with each other after sleep, because better veridical recall for a set of studied words may generalize to the corresponding critical lure (or vice versa). On the other hand, we propose that if sleep (versus wake) promotes gist abstraction, lure recall may become less related to or dependent on memories for the corresponding list items. This proposal is derived from iOtA's [9] prediction that sleep would selectively boost the overlapping gist memory (i.e. the lure) but not necessarily the studied words.

In this exploratory analysis, we first calculated a participant's number of correct recalls per DRM wordlist (Range = 0–8) and used this to predict recall of the corresponding critical lure in a generalized mixed-effect model, which had Number of intrusions, Number of correct recall per list, Interval (Immediate versus Delay), Test Time (AM versus PM), and interactions of the latter three variables as the fixed effects (see Appendix F for model output). There was a significant three-way interaction (*β* = 0.062, *z* = 2.80, *p* = 0.005), so we broke it down by computing two additional GLMMs, one within the Immediate and another within the Delay groups. These models had Number of intrusions, Number of correct recall per list, Test Time (AM versus PM), and an interaction of the latter two as the fixed effects. Table 6 summarizes the model outputs.

**Table 6.** Outputs from the exploratory generalized mixed-effect models examining the effects of intrusions, correct recall per list and Test Time in false recall.

fixed effects	immediate				delay			
	*estimate*	*s.e.*	*z*	*p*-value	*estimate*	s.e*.*	*z*	*p­*-value
Intercept	−3.681	0.153	−24.101	<0.001*	−3.593	0.154	−23.325	<0.001*
intrusions	0.070	0.010	6.854	<0.001*	0.023	0.007	3.257	0.001*
correct recall/list	0.656	0.030	22.033	<0.001*	0.955	0.042	22.694	<0.001*
Test Time	0.0219	0.0897	0.245	0.807	0.165	0.105	1.575	0.115
correct recall/list × Test Time	−0.001	0.026	−0.037	0.971	−0.127	0.036	−3.504	<0.001*

**Figure 6.**
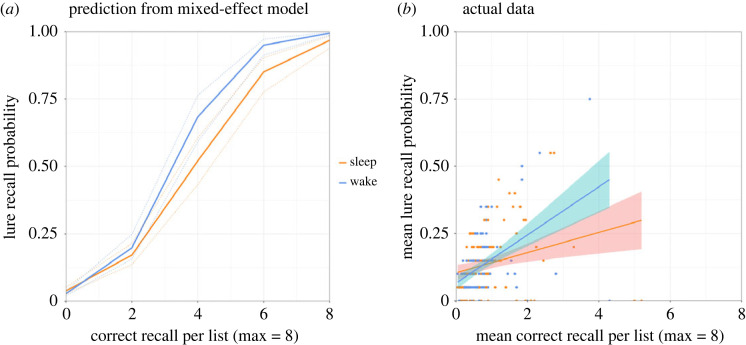
(*a*) Prediction from generalized mixed-effect model on the combined effects of correct recall/list and group (Sleep versus Wake) on false recall. (*b*) Correlation between lure and studied word recall rates in the Sleep and Wake groups. Each dot represents an individual participant. Note. Dotted lines/shaded areas represent 95% confidence intervals.

Across the Immediate and Delay groups, the number of intrusions and correct recall per list both had a main effect (*p*s ≤ 0.001) such that they were positively correlated with lure recall. However, the effect of Test Time was not significant for either group (*p*s > 0.11). Finally, the Correct recall per list × Test Time interaction was significant for the Delay (*z* = −3.504, *p* < 0.001) but not the Immediate groups (*z* = −0.037, *p* = 0.971). To interpret the former, we used the R package, ‘effects’ [108], to visualize it (figure 6*a*) and plotted a participant's lure recall probability against their veridical recall rates ( figure 6*b*).

Firstly, the positive correlations indicate that when a participant recalled more studied items from a DRM wordlist, they were also more likely to recall the corresponding critical lure, suggesting some kind of retrieval-induced generalization. Importantly, however, this effect was significantly *weaker* in the Sleep (versus Wake) group. This is striking, especially in light of our confirmatory findings that overall, the Sleep participants produced more critical lures (with intrusions controlled for) and more studied list items than their Wake counterparts. What this exploratory analysis suggests is that after sleep (versus wakefulness), whether a lure was recalled may be less reliant on the retrieval of its corresponding studied items (or vice versa). We contend that this provides preliminary evidence for our proposal that sleep may have impacted DRM false memory via gist abstraction processes, which make the lure more prominent and the studied list words less so. Note, however, that this did not rule out the possibility of other cognitive processes being at play.

#### 2.13.4. Semantic distance between intrusions and critical lures

As per the classic DRM literature, responses that were neither the studied list words nor the critical lures were classified as intrusions. For instance, our participants studied *nurse, sick, lawyer, medicine, health, hospital, dentist* and *physician*, with *doctor* as the critical lure. Responses such as *clinic* and *coconut* would both be considered intrusions, but clearly, *clinic* is semantically more related to the list items. In other words, there is much diversity within the intrusion data (e.g. [109]). Here, we explored whether our four groups differed in terms of the semantic distance between intrusions and critical lures, as indexed by pre-trained semantic spaces (ukWaC; [110]) derived from word2vec [111].^14^ We reasoned that since lure recall was greater in our Sleep (versus Wake) participants, their intrusions could potentially be more related to the lures in semantic space (e.g. [4]). To test this, we computed the cosine similarities between each intrusion and each of the 20 critical lures (table 7 for an illustration). The intrusion-lure pair with the highest cosine similarity (i.e. the nearest neighbour) was used for this analysis.

**Table 7.** Procedure for the exploratory analysis on semantic distance between intrusions and lures*.*

participant ID	intrusion produced by a participant	lure	cosine (in a descending order)		
1	ear	smell	0.405		
1		sleep	0.302		
1		doctor	0.239		
1		window	0.129		
.	↓				
**participant ID**	**intrusion produced by a participant**	**closest lure**	**cosine**	**number of intrusions produced**	**average cosine per participant**
1	ear	smell	0.405	4	0.347
1	metro	city	0.351		
1	heavy	slow	0.413		
1	clear	slow	0.219		
2	clinic	doctor	0.494	2	0.489
2	beach	mountain	0.484		

Since the number of intrusions varied greatly across participants, we averaged the lure-intrusion cosines on a participant level and used this as the dependent variable. A 2 (Interval) × 2 (Test Time) between-participant ANOVA revealed no effects of Interval (*z* = 2.541, *p* = 0.112) or Test Time (*z* = 0.085, *p* = 0.771), and their interaction was also non-significant (*z* = 0.886, *p* = 0.347). We explored further by comparing the cosines between the Sleep and Wake groups. While this comparison is in the predicted direction (*M*Sleep = 0.381, s.d.Sleep = 0.072 versus *M*Wake = 0.370, s.d.Wake = 0.085), it was not statistically significant according to an emmeans pairwise comparison (*z* = 0.874, *p* = 0.383). In sum, while the Sleep (versus Wake) groups produced fewer intrusions overall, we found no evidence that their intrusions differed in the degree of semantic relatedness.

## 3. Results summary

To help the reader gain a better understanding of the overall picture, table 8 summarizes our key findings.

**Table 8.** Summary of key findings. Note. NS, not significant, * = *p* < 0.05, ** = *p* < 0.01, *** = *p* ≤ 0.001, n.a., not applicable (not tested).

dependent variable	Interval × Test Time	*M*_AM_	*M*_PM_	pairwise comparison	*M*_Sleep_	*M*_Wake_	pairwise comparison	interpretation
**confirmatory**								
intrusions	**	4.67	5.62	***	6.78	10.10	***	(a) clear time-of-day effect such that evening testing resulted in more intrusions (b) Above and beyond this effect, there were fewer intrusions after sleep
critical lures (Max = 20)	*	2.41	2.69	NS	2.73	2.51	*	greater false recall in the Sleep group, partly reflecting a greater lure-to-intrusion ratio after sleep
studied list words(Max = 160)	***	21.18	24.36	NS	17.33	13.41	***	greater veridical recall after sleep
**exploratory**								
total responses	NS	28.25	32.67	n.a.	26.83	26.01	n.a.	sleep and wake groups were well-matched

## 4. General discussion

To-date, about ten published studies (e.g. [32,33,65]) have asked how overnight sleep (versus daytime wakefulness) may influence false and veridical memories in the DRM paradigm. Despite these prior attempts, the existing evidence base was contradictory. A recent meta-analysis [46] attempted to reconcile the literature and identified list length as a potential moderator such that sleep may increase false memory when a DRM list contains fewer related words. Motivated by this finding, our registered report tested 488 participants, who studied short DRM lists (i.e. 8 words/list) and completed free recall either shortly afterwards (AM-Control & PM-Control) or after a 12 h delay containing overnight sleep or daytime wakefulness. Our registered report represents the most highly powered study to-date to examine how sleep (versus wake) influences DRM false (and veridical) memories, providing a firm empirical base for theoretical development.

Our confirmatory frequentist analyses found evidence of the Sleep (versus Wake) participants producing fewer intrusions, above and beyond any time-of-day effects. They also recalled more studied list words. Importantly, when the number of intrusions was statistically controlled for, the Sleep participants falsely produced more critical lures. An exploratory analysis showed that this partly reflects a greater lure-to-intrusion ratio after sleep. Thus, our overall findings suggest that sleep may have had the effect of increasing both veridical and DRM false memories while reducing intrusions. Another way of describing this pattern is that sleep appears to benefit both (i) the accuracy of participants' memory for the word lists (correct veridical recall), plus (ii) the gist-like nature of the errors (fewer arbitrary intrusions and more critical lures). These effects were seen in the context of no difference between the sleep and wake groups in the number of total responses. We interpret these as potentially suggesting that sleep may have boosted two inter-related mechanisms: (1) gist abstraction/spreading activation and (2) memory suppression/source monitoring. We expand on this interpretation in the following sections.

Surprisingly, our complementary Bayesian analyses revealed moderate-to-strong evidence for the null hypotheses in both veridical and false recall, rendering interpretation of our findings less straightforward than expected. However, it is worth noting that while the current study met the power requirements for our frequentist analyses, it is unclear if it met those for a properly powered Bayesian analysis (e.g. [71]). Therefore, in keeping with our pre-registration, we base our interpretation on the outcomes of our frequentist analyses while adopting a cautious stance.

### 4.1. How do our findings of sleep increasing false and veridical recall but reducing intrusions sit with extant theories?

#### 4.1.1. Lo *et al*. Fenn *et al*.'s theories

Our finding of greater false recall post-sleep (with intrusions controlled for) seems to contradict Lo *et al*.'s [3] theory, which is argued to be an extension of the synaptic homeostasis hypothesis [52,53]. Specifically, Lo and colleagues proposed that peripheral aspects of an encoded memory (e.g. sensory details of a studied list word) would be pruned during sleep-related consolidation, improving accessibility for the list words, and in turn, aiding suppression of the critical lures at test. Lo *et al*.'s interpretation argues for a post-sleep *reduction* in DRM false memories, which is at odds with our false recall data. Similarly, our finding also argues against Fenn *et al*.'s [32] interpretation of a standard active consolidation theory, who proposed that sensory details associated with a studied list word may be better consolidated over sleep (versus wake), which may, in turn, facilitate suppression of the lures. Again, our results do not support this proposal.

While our false recall data do not support a key prediction from Fenn *et al*. and Lo *et al*.'s theories, our data cannot dismiss them entirely. This is because our Sleep group produced significantly fewer intrusions than the Wake group. This finding has some conceptual fit with Fenn *et al*. and Lo *et al*.'s theories that sleep may enhance source monitoring/memory suppression and hence reduce the incidence of incorrect information (including both lures and intrusions).^15^ Therefore, these theories do have some validity in the context of our intrusion (and veridical recall) data, despite not being able to explain our greater lure-to-intrusion ratio after sleep. Potentially, as discussed in greater detail below, while sleep may benefit source monitoring/memory suppression, the effect of sleep may extend to other processes such as gist abstraction and spreading activation, providing a plausible explanation to our overall findings.

Returning to the evidence base on critical lures, we should note that our results (using recall) are at odds with some previous studies finding that sleep (versus wake) reduced false recognition of the critical lures (e.g. [32]). Could recall and recognition tests lead to opposite effect of sleep? We think that this is unlikely. First, it is worth pointing out that both Monaghan *et al*. [76] and Shaw & Monaghan [64] found that post-encoding sleep (versus wakefulness) enhanced false recognition in young adults. Moreover, a study [113] that came out during the preparation of this manuscript again showed that sleep increased DRM false recognition (although participants encoded the wordlists under a more incidental condition). These later results suggest that the evidence for sleep reducing false recognition is rather mixed. Second, while sleep-related memory effects are known to be larger in recall than in recognition (see [73] for a review), the effects of sleep in these memory tests rarely, if ever, pattern in *opposite* directions. Relatedly, a wealth of ‘standard' DRM studies that did not manipulate sleep (e.g. [31,68,114,115]) investigated how false recall and recognition may be modulated by various variables, from list length to personality. To the best of our knowledge, the effect of these variables always patterned in the same direction across recall and recognition. Given these different strands of evidence, we hold reservations over early empirical findings of sleep (versus wake) reducing false recognition, at least in young adults (see Scullin & Bliwise [116] for a review on older adults).

#### 4.1.2. Fuzzy-Trace theory

This theory argues that studying a DRM wordlist results in two memory traces: a verbatim and a gist trace. It is not a sleep theory *per se* and has no clear prediction for how sleep may affect these traces. As outlined in the introduction, we see multiple possibilities: Sleep (versus Wake) may boost both the verbatim and gist traces (1) equally, or (2) at varied strength, (3) selectively the verbatim trace, or (4) selectively the gist trace. Below, we consider these possibilities in turn.

We found moderate evidence that the Sleep (versus Wake) participants had better veridical recall, greater lure recall (with intrusion controlled for), and produced fewer intrusions. This overall pattern appears consistent with the possibility that both the gist and verbatim traces were enhanced by sleep-related memory processes. On one hand, sleep (versus wake) may have benefitted gist abstraction, increasing the relative incidence of lure recall. On the other hand, sleep may have facilitated the retention and consolidation of studied list words, resulting in enhanced veridical recall and greater suppression of intrusions. Currently, we cannot determine whether the verbatim and gist traces were equally enhanced or if one was influenced more strongly by sleep. However, exploratory analysis #2 revealed that when the number of intrusions was removed from our confirmatory analyses, the sleep versus wake pairwise comparison remained statistically significant for veridical recall, but not for lure recall. Potentially, this suggests that the effect of sleep may have been more direct and/or stronger for the verbatim than for the gist trace.

Moving on, our overall findings seem to rule out possibility (3) (i.e. verbatim trace being selectively enhanced), because if verbatim traces were selectively enhanced during sleep-related processes, the critical lures should have been better suppressed via some suppression/monitoring mechanisms (e.g. [117,118]), leading to a post-sleep reduction in lure recall (e.g. [32]), which we did not find.

Our final possibility was that sleep leads to the gist trace being selectively strengthened. According to FTT, false and veridical recall can be based on the same gist representations [42]. Therefore, it is plausible that sleep-related processes selectively enhanced the gist trace, simultaneously increasing lure and veridical recall. However, if sleep selectively enhanced the gist trace, there should be a post-sleep increase in intrusions as well, especially thematically related ones (e.g. *clinic* for the *doctor*-list), because gist traces are more error-prone than verbatim traces [119]. Contrary to this, our sleep (versus wake) participants produced *fewer* intrusions, and we found no evidence that their intrusions were more thematically related to the lures (see exploratory analysis #4). These make it hard to argue that sleep selectively benefitted the gist trace and had no influence on the verbatim trace.

#### 4.1.3. Activation/monitoring framework

This framework, which is also not a sleep-specific theory, assumes two cognitive processes: spreading activation within associative networks and source monitoring that aids memory suppressions. Some prior evidence suggests that post-encoding sleep (versus wakefulness) may benefit spreading activation ([47,48]; but see [50]), and in line with this, there was moderate evidence of greater lure recall post-sleep (with intrusion controlled for), suggesting that sleep may have increased activation spreading into (or being maintained within) the critical lures. Interestingly, however, if sleep solely promoted spreading activation, more unseen (but related) words would have been produced by the participants, increasing the number of intrusions. However, our sleep (versus wake) group produced *fewer* intrusions, so potentially, in addition to promoting spreading activation, sleep may have also benefited source monitoring to a certain degree (e.g. [32]), preventing activation from spreading too far away from the lures/studied list words.

Finally, regarding veridical recall, the Activation/Monitoring framework may make different predictions, depending on the assumptions in place. Our findings of better veridical recall post-sleep argue that sleep (versus wake) may have (i) increased the amount of activation circulating within an associative network, and/or (ii) enhanced monitoring processes, thereby preventing activation from diffusing beyond the network. This would explain why sleep enhanced veridical recall but reduced intrusion rates.

#### 4.1.4. iOtA

Unlike the previous two theories, iOtA is a sleep theory that explicitly predicts a post-sleep increase in DRM false memory [9]. It proposes that individual memories, such as the studied list words, would be reactivated during sleep, and their overlapping areas (i.e. the critical lure) would be selectively consolidated. We found moderate evidence of greater lure recall post-sleep (with intrusions controlled for), in alignment with iOtA's overarching prediction. Furthermore, in exploratory analysis #3, we found a weaker correlation between veridical and lure recall after sleep (versus wake). We propose that this is suggestive of sleep-related gist abstraction processes such that during sleep, a broader conceptual understanding of the DRM wordlists emerged or became more prominent, but at the same time, specific details of individual words became less important. This possibility, in our view, conceptually fits with the iOtA framework, which emphasizes a role of sleep in strengthening the shared elements of individual memories.

There were, however, two aspects of our findings that do not readily align with the iOtA framework. First, it is not immediately clear how this framework accommodates the finding of our sleep (versus wake) participants producing fewer intrusions. Potentially, combining iOtA with a source monitoring/suppression theory, as described in previous sections, could be a fruitful way forward. Second, the iOtA framework does not provide a straightforward explanation for our veridical recall data. As outlined in our introduction, iOtA predicts that sleep has limited effects on the non-overlapping elements themselves (i.e. the studied list words), so it is not immediately clear how iOtA explains the increase in veridical recall post-sleep. Potentially, iOtA may appeal to the possibility that a night of sleep selectively benefitted the gist trace, leading to a concurrent boost in false and veridical recalls [120]. However, as explained in the section on FTT earlier, our intrusion data suggest that sleep does have an effect on the verbatim trace. Therefore, to accommodate the simultaneous increase in false and veridical recalls post-sleep, iOtA needs further tightening and may consider the possibility that gist abstraction and veridical memory consolidation occur alongside each other, at least in the first post-encoding sleep.

#### 4.1.5. Interference theory

One of the explanations for how sleep benefits declarative memories involves the reduction of interference, such that sleep protects encoded memories from sensory/linguistic input during wakefulness [12,15,16]. Such an account struggles to explain our lure recall data, as it predicts that sleep should reduce interference and, therefore, lead to significantly fewer critical lures being produced. While an interference account falls short in addressing our lure recall data, it can easily explain our findings of reduced intrusions (see §4.2) and enhanced veridical recall post-sleep. As such, there are some merits in an interference account, and it is likely that interference reduction contributes to some aspects of our findings.

#### 4.1.6. Summary

In this section, we considered three sleep-specific theories: (1) Fenn *et al*.'s [32], (2) Lo *et al*.'s [3], and (3) the iOtA framework [9]. Our false recall data are not consistent with a key prediction of theories (1) and (2) but are in line with that of (3). Interestingly, our post-sleep reduction in intrusions conceptually aligned with theories (1) and (2) but cannot be easily explained by theory (3). In short, while these sleep-specific theories have their strengths, we argue that they cannot fully capture our overall findings and that a combination of these theories may be a fruitful way forward.

Beyond the three sleep-specific theories discussed, we also considered two general theories that are not sleep-specific: FTT and Activation/Monitoring Framework. Both display potential in accommodating all our findings. In the context of FTT, our findings may be explained if sleep benefits both the verbatim and gist traces (but perhaps to a different degree). And for the Activation/Monitoring Framework, we argued that a combination of greater spreading activation and source monitoring could explain our findings. Therefore, our study provides a new and valuable test case for refining the contribution of sleep in these general theories.

### 4.2. Time-of-day effects in intrusions

Rather unexpectedly, and contrary to the null findings from prior ‘Sleep × DRM' studies (e.g. [33,34]), participants who completed free recall in the evening (i.e. the PM-control and Wake groups) produced more intrusions than those in the morning (i.e. the AM-control and Sleep groups). Remarkably, Test Time exhibited no significant and consistent effect on either total responses or lure/studied word recalls, implying a relatively specific effect. Also worth noting is that our four groups were well-matched on their degree of sleepiness and circadian preference (table 3), implying that the effect of Test Time on intrusions is unlikely to be due to differences in alertness, which can impact performance in some cognitive tasks (e.g. [121–123]). As to why evening (versus morning) testing led to a selective increase in intrusions, we propose that it might be related to interference from sensory/linguistic input accumulated throughout the day.

Participants in the PM and Wake groups should have accumulated a fair amount of sensory/linguistic input in the 10–12 h leading up to free recall, while those tested in the morning (AM and Sleep groups) should have accumulated less due to sleep. These morning participants may also benefit from one of the proposed functions of sleep, which is to ‘reset' the brain by pruning (relatively unimportant) information accrued prior to sleep [52,53]. Therefore, it seems reasonable to infer that participants tested in the evening (versus morning) may have experienced more interference from sensory/linguistic input, which may have, in turn, weakened source monitoring/memory suppression and thus increased in intrusions at retrieval.

### 4.3. Advancing the evidence base for Sleep × DRM studies

To test for a sleep effect in the DRM paradigm, some prior studies conducted two statistical tests (e.g. separate *t*-tests), one comparing Sleep versus Wake, another comparing AM- versus PM- controls (e.g. [32,33]). It was then concluded that sleep had a unique effect that extends beyond time-of-day influences when the Sleep versus Wake comparison was statistically significant (*p* < 0.05) but not the AM versus PM comparison (*p* > 0.05). However, as cautioned by Gelman and Stern [78] and Nieuwenhuis *et al*. [77], this is not sufficient as the distinction between ‘significant' and ‘not significant’ lacks statistical significance in itself. Our study advances the ‘Sleep × DRM' literature by showing significant interactions between Interval and Test Time, providing compelling evidence against our sleep-related findings being primarily driven by time-of-day effects. This strengthens the robustness of our conclusions and emphasizes the distinct influence of sleep in the DRM paradigm.

In addition to demonstrating significant Interval by Test Time interactions, our study critically showed that participants in the Sleep and Wake conditions differed in their response quality even though they were matched on response quantity (as indexed by total responses) (see also [4] for relevant findings). Specifically, the Sleep group produced fewer responses that were unlikely to be useful for future memory tests (i.e. intrusions) but more responses that can be seen as beneficial (i.e. studied list words & critical lures as they represent the gist). Potentially, these reflect the possibility that sleep may have boosted two interrelated mechanisms, gist abstraction/spreading activation on one hand and memory suppression/source monitoring on the other. Our findings are generally consistent with the view that sleep may serve a broader purpose beyond the protection of declarative memories. A small but growing literature has suggested that sleep may transform and reorganize memory, enabling the generation of insights and abstractions [18,124–126], the formation of inferences [127], and their integration into pre-existing semantic networks [128]. However, it is clear from both our lure recall data and recent sleep studies (e.g. [129,130]) that substantial transformation is unlikely over the course of a single night of sleep. It is more likely that gradual transformation takes place over the course of many periods of sleep (e.g. weeks or months).

Finally, our exploratory analysis on the correlation between veridical and lure recall is also illuminating. As far as we are aware, we are the first to demonstrate a weaker correlation between veridical and lure recall post-sleep (versus post-wakefulness). This finding suggests that after sleep, recall of the critical lures might have become less dependent on whether their respective list items were remembered. We propose that this potentially reflects sleep-related gist abstraction processes such that sleep facilitates a gradual shift towards a broader conceptual understanding of the material, where the specific details of individual items matter less. This possibility warrants further research and holds promise to enhance our understanding of memory transformation during sleep.

### 4.4. Limitations

Despite the valuable insights provided by this research, it is essential to acknowledge several limitations inherent in our study.

First, our experiment was conducted online, potentially introducing variations in participant engagement, distractions, and environmental conditions compared to in-person settings. To alleviate the potential issues this may bring, we requested participants to provide information on their surrounding environment in the test phase survey (see Appendix D), although we did not formally analyse these factors. Despite being conducted online, our study showed clear evidence for the classic DRM false memory effect and a sleep-related benefit in veridical recall. Furthermore, we observed a typical U-shaped serial position curve (e.g. [84,106]) in veridical recall (further info available on OSF). All these offer reassurance of our data quality. In fact, administering the experiment online, while losing control over some variables, provides potential advantages in other respects. For example, it mirrors how most participants typically encode information in real life. This enhances the ecological validity of our study.

Second, to ensure comparability with prior ‘Sleep × DRM' studies (e.g. [33]), we recruited exclusively young adults (aged 18–25) for our study. However, this means that our findings may not be applicable to different age groups.

Additionally, we used free recall as the sole outcome measure, so it is unclear whether our findings extend to recognition. However, as indicated by Newbury & Monaghan's [46] meta-analysis, sleep had an even smaller effect size on false recognition than on false recall. Considering the modest-to-moderate sleep effect we observed in false recall, it begs the question of how practically and theoretically meaningful it is to investigate the effect of sleep on DRM false recognition.

Lastly, even though our participants were randomly assigned to the four experimental groups, there could still be inherent biases due to self-selection. For example, individuals with a morningness (versus eveningness) preference might have been more inclined to participate in the Immediate-PM/Wake groups (e.g. [85]; Experiment 1). Fortunately, our analysis showed that the four experimental groups were well-matched in terms of sleepiness ratings and morningness/eveningness preferences ( table 1), suggesting that self-selection had a minimal effect on our results.

## 5. Conclusion

Our registered report assessed free recall for short DRM wordlists in young adults who had an overnight sleep opportunity (versus engaging in normal daytime activities) in the 12 h interval between study and test. The results suggest an intriguing combination of effects. The Sleep and Wake groups were well matched in the number of total responses after the 12 h delay. Despite this, the Sleep participants were more accurate in their veridical memory of the studied list words as well as more gist-like in their incorrect responses (a greater lure-to-intrusion ratio). Sleep-specific theories such as the iOtA framework are able to explain some but not all of our findings, suggesting that theoretical tightening or an alternative approach is needed. By contrast, two more general theories, FTT and Activation/Monitoring Framework (AMF), could conceivably provide a satisfactory explanation, but they are silent on the role of sleep (versus wake). Considered in the light of these frameworks, our study provides a rich new body of evidence to help determine the contribution of sleep. Overall, our findings point to sleep potentially boosting (1) gist abstraction and memory suppression (FTT) and/or (2) spreading activation and source monitoring (AMF). Furthermore, an exploratory analysis showed, for the first time, that lure recall was less dependent on studied word recall after sleep. Speculatively, this may reflect a drive towards gist-like representations emerging or becoming more prominent after sleep. In summary, our registered report not only helps reconcile the existing ‘Sleep × DRM' literature, but also stands as a significant stride towards understanding the role of sleep beyond memory retention.

## Data Availability

This article has no additional data.

## Footnotes

1 We explored this potential moderator in an exploratory analysis (available on OSF).

2 In our Stage-1 proposal, we proposed that the study and test phases would start at 9:30/21:30 (±1 h). However, due to an oversight, both phases started half an hour earlier, at 9:00/21:00 (±1 hr) instead.

3 Prior studies in the ‘Sleep×DRM' literature (e.g. [32,33]) conducted two separate statistical tests, one comparing Sleep versus Wake, another comparing AM- versus PM- controls. They then concluded that Sleep had an effect on DRM false memory beyond time-of-day effects when Test Time was significant in the Sleep versus Wake comparison (*p* < 0.05) but not in the AM versus PM comparison (*p* > 0.05). Unfortunately, however, this is not sufficient [77], as ‘the difference between ‘significant' and ‘not significant' is not itself statistically significant' [78]. Therefore, in order to rule out time-of-day effects, one needs to show that Sleep versus Wake is significantly different from AM versus PM-control. This can be captured by an Interval × Test Time interaction.

4 Newbury and Monaghan [46] found no evidence in their meta-analysis that the modality of presentation modulated the effect of sleep. However, in a set of three experiments, Fenn *et al*. [32] used auditory presentation in one of them and visual in the other two. As far as we are aware, this is the only study in the ‘Sleep × DRM' literature that had used both modalities in the same set of experiments. Sleep appeared to have a larger effect on false memory when visual (versus auditory) presentation was used. Given this, we opted for visual presentation in the current experiment.

5 Contrary to our a priori prediction, the distribution of veridical recall had a clear positive skew (Range = 1–104; Median = 16; Mean = 19.13; Shapiro-test: *p* < 0.001), making standard deviation an undesirable measure of variability. Instead of standard deviation, we could use e.g. interquartile range. However, our pre-registration did not specify a threshold when an alternative is to be used (e.g. 1.5 or 3 IQR), so we were reluctant to go down the route of setting an arbitrary threshold post hoc. Furthermore, the intended purpose of this exclusion criterion was to exclude participants who might have cheated by writing down all the words (e.g. achieving near-ceiling accuracy). However, even the best performer only recalled 104 (or 65%) of the studied list words, so we are inclined to believe that no cheating had occurred. We, therefore, decided to remove this exclusion criterion and not to replace it.

6 If a misspelt response has more than one nearest word, the response was considered as an intrusion.

7 The use of GLMM is a clear departure from prior ‘Sleep × DRM' studies, the majority of which addressed the same research question using an independent *t*-test or ANOVA (e.g. [33,65,76]). We explained in **Appendix E** why these statistical tests are usually not appropriate in the context of DRM recall and why GLMM are more advantageous.

8 To obtain the Bayes Factor for the simple effects of Test Time, the alternative model will contain Test Time as the sole fixed effect while the null model will contain no fixed effects.

9 Outcome: our free recall data demonstrated a clear U-shaped serial position effect such that the first (1st) and last (8th) items in the DRM wordlists were better recalled than those in the middle.

10 Outcome: inconsistent with Diekelmann *et al*. [65], we found no evidence that the effect of sleep on false recall was modulated by adjusted veridical recall.

11 Since some participants produced 0 lures/intrusions, we added a constant of 0.1 to all values before computing the lure-to-intrusion ratios.

12 Retrieval-induced generalization: retrieval of one word cueing retrieval of a related word (e.g. [107])

13 Gist abstraction: extraction of the central or essential meaning of learned information.

14 We additionally used semantic spaces derived from Latent Semantic Analysis [112], and the results are essentially the same as those reported here (i.e. using word2vec).

15 We thank Dr Michael Scullin for pointing this out.

## Open science statement

All the materials, data and analysis scripts are publicly available on Open Science Framework (https://osf.io/9pdyf/), which also hosts a fully reproducible RMarkdown file of this paper (kindly provided by Dr Daniel Baker).

## Appendix A. Hypotheses, sampling plan, analysis plan and interpretation given each statistical outcome for each of the questions of theoretical interests.

**Table:**

question	hypothesis	sampling plan	analysis plan	rationale for deciding the sensitivity of the test for confirming or disconfirming the hypothesis	different outcomes+interpretations	theory that could be shown wrong by the outcomes
Does overnight sleep (versus daytime wakefulness) influence DRM false recall?	Sleep will increase DRM false recall.	We simulated a dataset that approximated the data distribution of a prior study ([33]; Experiment 1). We then manipulated the data so that it fits with our effect size assumptions. After 500 Monte Carlo simulations, we found that 480 participants in total will give over 85% power to detect a significant interaction between Interval and Test Time as well as a simple effect of Sleep versus Wake.	A generalized linear mixed effect model will be fitted to the false recall data using the lme4 and buildmer packages. The dependent variable is binary: whether a critical lure is recalled or not (1 versus 0). The fixed effects will be Interval (Immediate versus Delay), Test Time (9:00 versus 21:00), and their interaction. Then, we will test the simple effects of Test Time within the Immediate and Delay groups using the emmeans package.	Effect sizes were informed by a recent meta-analysis [46]. We opted for the lower-bound of the 95% confidence intervals reported.	**Outcome 1:**	This will argue strongly against theories (e.g. synaptic homeostasis hypothesis) that predict greater suppression of false memory after sleep.
					A significant interaction between Interval and Test Time	
					A significant simple effect of Test Time within the Delay groups, where Sleep > Wake	
					With or without a simple effect of Test Time within the Immediate groups.	
					→ Supports theories (e.g. iOtA and spreading activation) that predict a role of sleep in promoting gist abstraction and/or spreading activation.	
					**Outcome 2:**	This will argue against theories (e.g. iOtA) that predict a specific role of sleep in promoting gist abstract and spreading activation.
					A significant interaction between Interval and Test Time	
					A significant simple effect of Test Time within the Delay groups, where Sleep < Wake.	
					With or without a simple effect of Test Time within the Immediate groups.	
					→ Supports theories that predict greater suppression of DRM false memory after sleep	
					**Outcome 3:**	We will not be in a position to falsify any theories.
					A significant OR non-significant Interval×Test Time interaction	
					A non-significant simple effect of Test Time within the Delay groups	
					→ Sleep did not have a significant effect on DRM false recall. We cannot rule out the possibility that this null finding is due to our study being conducted online; however, this raises the question of how robust prior findings are, which were mostly based on small sample sizes and questionable statistical methods.	
					**Outcome 4:**	We will not be in a position to falsify any theories.
					A non-significant interaction between Interval and Test Time	
					A significant simple effect of Test Time within the Delay groups, where Sleep > Wake or Sleep < Wake	
					A significant or non-significant simple effect of Test Time within the Immediate groups	
					□While false recall differed between the Sleep and Wake groups, we cannot rule out the possibility that this difference is due to time-of-day effects. Notably, almost all prior studies did not test for an interaction between Interval and Test Time, so this pattern of results would suggest that their findings might have been confounded by time-of-day effects.	
Does overnight sleep (versus daytime wakefulness) increase veridical recall of the studied list words?	Sleep will increase veridical recall.	The same as Q1	A generalized linear mixed effect model will be fitted to the veridical recall data using the lme4 and buildmer packages. The dependent variable is binary: whether a studied list word is recalled or not (1 versus 0). The fixed effects will be Interval (Immediate versus Delay), Test Time (9:00 versus 21:00), and their interaction. Then, we will test the simple effects of Test Time within the Immediate and Delay groups using the emmeans package.	This was based on Q1.	**Outcome 5:**	NA
					A significant interaction between Interval and Test Time	
					A significant simple effect of Test Time within the Delay groups, where Sleep > Wake. (note that Sleep < Wake is very improbable given a decade of prior evidence).	
					→ Interpretations will depend on the outcome for the previous research question	
					(a) *In combination with Outcome 1 above (*i.e. *Sleep increases false recall)*	
					→ Sleep-related consolidation may have helped stabilize and strengthen both the verbatim and gist traces (or both list words and lure activation). Alternatively, sleep may have boosted the gist traces/lure activation, which in turn increased both veridical and false recall. Theories that predict a selective increase in false recall post-sleep (e.g. iOtA) will need adjustments.	
					(b) *In combination with Outcome 2 above (*i.e. *Sleep reduces false recall)*	
					→ False memories may have been better suppressed after sleep because sleep boosted veridical memories. This will provide support for theories that proposed a role of sleep in influencing sensory details of list words [3,32].	
					(c) *In combination with Outcome 3 or 4 above (*i.e. *Insufficient evidence for a sleep effect in false recall)*	
					□ While there is no evidence that a night's sleep influences false recall, sleep benefits veridical memory (e.g. [16]).	
					**Outcome 6:**	
					A non-significant interaction between Interval and Test Time+A significant simple effect of Test Time within the Delay groups	
					OR	
					A non-significant simple effect of Test Time within the Delay groups, regardless of whether the Interval×Test Time interaction is significant	
					→ Insufficient evidence for a role of sleep in veridical recall, but precise interpretations will depend on the outcome for the previous research question (see below).	
					(a) *In combination with Outcome 1 above (*i.e. *Sleep increases false recall)*	
					→ A sleep effect may be selective in the sense that sleep benefits gist abstraction/lure activation but not necessarily individual memories. This would support the iOtA model.	
					(b) *In combination with Outcome 2 above (*i.e. *Sleep reduces false recall)*	
					→ Reduction of false memory may not necessarily be a result of a post-sleep increase in veridical memories, which has been hypothesized to enhance suppression of false memory (e.g. [3,32]).	
					(c) *In combination with Outcome 3 or 4 above (*i.e. *Insufficient evidence for a sleep effect in false recall)*	
					→ A night's sleep has no significant effect on either veridical or false memories in the DRM paradigm. We cannot rule out the possibility that this null finding is due to our study being conducted online; however, this raises the question of how robust prior findings are, which were mostly based on small sample sizes and questionable statistical methods.	

## Appendix B. *A priori* power analysis

According to Newbury & Monaghan's [46] meta-analysis, the effect size for sleep in DRM false memory is Hedge's *g* = +0.92 (95% CI: 0.54, 1.30; *p* < 0.001) when short lists (10 words per list) were used.^16^ However, due to certain biases in the psychology literature (e.g. publication bias), it has been argued that published effect sizes are generally inflated (e.g. [133]) and that power analysis should be based on the lowest meaningful estimate [134,135]. Therefore, we opted for a more conservative (yet contextualized) effect size estimate and went for the lower-bound of the 95% confidence interval reported by Newbury & Monaghan [46], which is *g* = +0.54.

On estimating power in GLMM, a recent guideline [136] recommends using well-powered data from previous experiments. However, as far as we are aware, no prior ‘Sleep × DRM' studies to date have made their datasets publicly available. We, therefore, simulated a dataset for our power calculation (available on OSF).

The first step is to determine a sample size. We have the financial resources to reach up to 160 participants/group (i.e. 640 in total), so we began by fabricating a dataset containing 120 participants/group. We made up 20 false recall observations for each participant. We then split the dataset by Interval, so one dataset for the Delay (Sleep+Wake) groups, another for the Immediate (AM + PM-control) groups, with each containing 4800 observations from 240 participants. In the first dataset, we simulated the false recall data for the Delay groups such that they approximated the data distribution [*M*_Sleep_ = 45.9% (s.d. = 20.6%) versus *M*_Wake_ = 36.3% (s.d. = 21.2%), *p* = .005] from a prior study ([33]; Experiment 1). Then, the data were manipulated to fit with our effect size assumption, such that the effect size for sleep is *d* = 0.54 [*M*_Sleep_ = 44.7% (s.d. = 12.6%) versus *M*_Wake_=38.0% (s.d.=12.0%); *t*_237.4_ = 4.20, *p* = 0.001]. Afterwards, we simulated the false recall data in the second dataset for the AM and PM-control groups such that they also approximated the data distribution in Payne *et al*. [33] versus *M*_PM_ = 46.3% (s.d. = 23.5%), *p* = 0.57) and that they did not differ significantly from each other [*M*_AM_ = 42.9% (s.d. = .0%) versus *M*_PM_ = 43.5% (s.d. = 14%); *t*_233.3_ = −0.37, *p* = 0.709, *d* = *−*0.05].^17^

We then merged the two fabricated datasets together and fitted a GLMM to it, using the lme4 package [137]. The dependent variable, fixed effects structure, coding scheme and computation procedures were identical to those in our confirmatory analysis (see section 12.3). Table 9 shows the fixed-effects estimates from the converged model, which has a by-participant intercept only.

**Table 9.** Fixed-effects estimates from the converged maximal model examining the effects of Interval and Test Time in the fabricated dataset.

fixed effects	estimate	s.e.	*z*	*p*-value
intercept	−0.316	0.024	−13.24	<0.001
Interval	−0.039	0.024	−1.65	0.099
Test Time	0.063	0.024	2.663	0.007
Interval × Test Time	0.076	0.024	3.187	0.001

Based on the fixed-effects estimates, we estimated the power a sample size of 480 (i.e. 120/group) has for detecting an Interval and Test Time interaction. We conducted Monte Carlo simulation using the ‘simr' package [138] in R (see box 2 for R codes). After 500 simulations, it was estimated that a sample of this size gives 90.1% power (95% CI: 87.03, 92.49) to detect a significant interaction. Then, we estimated the power we have for detecting a simple effect of Test Time within the Delay groups (i.e. Sleep versus Wake). Following the simulation procedures above, it was estimated that 120 participants/group (i.e. 240 in the Delay groups) will give about 98.8% power (95% CI: 97.41, 99.56). In sum, our power calculation showed that having 120 participants/groups will give ample power (greater than 90%) to detect both an Interval × Test Time interaction and a simple effect of Sleep versus Wake. Finally, we also estimated that we will have at least 80% power as long as we have greater than 99 participants/group. Therefore, in case we fail to reach our target sample size of 120 participants/group before funding expires but manage to recruit greater than 99/group, our proposed experiment will still have satisfactory power.

Box 2. R codes for Monte Carlo simulation. >library(simr) >fixef(fabricated_model)[‘Interval1:Test_Time1’] < −0.076 >set.seed(99) >powerSim(fabricated_model, fixed(Interval1:Test_Time1), nsim = 500) Power for predictor ‘Interval1:Test_Time1', (95% confidence interval):  90.00% (87.03, 92.49) Based on 500 simulations, (0 warnings, 0 errors) alpha = 0.05, nrow = 9600

We note that the focus of our proposed experiment is Research Question #1 [Does sleep (versus wakefulness) influence DRM false recall?], so we based our power analysis on this question. Despite this, the estimate of 120 participants/group will also give over 90% power to detect the desired effects for Research Question #2 [Does sleep (versus wake) increase veridical recall?], assuming the effect size for sleep is similar between Questions #1 and #2. Furthermore, since there are more studied list words than critical lures (160 versus 20), the GLMM for addressing Question #2 will have substantially more observations than that for Question #1 (approx. 76 800 versus 9600), boosting power on the item level. In short, our target sample size of 120 participants/group will give us sufficient power for both Research Questions.

## Appendix C. *Screening survey*

**Table:**

Page 1: Demographic information
1) What is your gender identity?
2) How old are you?
3) In what country do you currently live?
4) What is your first language(s)?
5) What is your ethnicity?
6) What is the highest level of education you have completed?
7) Do you have any history of any psychiatric (e.g. schizophrenia), developmental (e.g. autism, dyslexia) or sleep disorders (e.g. insomnia)?
8) If your answer to the above is Yes, please can you name the diagnosis/es?
Page 2: Outline of the main study
**IMPORTANT: Please read carefully**
We are recruiting hundreds of participants for a simple memory study. We would like to see if you may be interested in taking part.
In **Task 1**, participants will see and remember some English words. This will take about 10 min.
In **Task 2**, participants will complete a simple memory test based on the words they saw in Task 1. This requires about 12 min.
Participants will receive **£3.5** (£9.5/hour) upon completion of the two tasks.
Importantly, participants will be **randomly** allocated to one of the four groups:
Group A (AM Group): You can start Task 1 and 2 any time between 8:30 and 10.30.
Group B (PM Group): You can start Task 1 and 2 any time between 8.30 and 22.30.
Group C (Delay Group 1): You can start Task 1 any time between 8.30–10.30 and then Task 2 between 8.30–22.30 **on the same day.**
Group D (Delay Group 2): You can start Session 1 any time between 8.30–22.30 and then Task 2 between 8.30–10.30 **the day after.**
**Those in Groups C and D will receive a £0.2 bonus upon completion of the study.** Unfortunately, we are NOT able to accommodate any preferences for group allocation.
If you are happy to take part in our memory study, press Yes below. If not, press No.
Yes / No

## Appendix D. Survey in the test phase

**Table:**

1.	(Sleep group only) Approximately what time did you go to bed last night?
2.	(Sleep group only) Approximately what time did you wake up this morning?
3.	(Sleep group only) How would you rate the quality of last night's sleep?
	**Very Good, Good, Fair, Poor, Very Poor**
4.	(Wake group only) Did you have a nap between Session 1 and now?
	**Yes, No**
5.	(Wake group only) If your answer to the above is Yes, how long was the nap?
6.	Did you consume any alcoholic drinks in the last 12 h? If Yes, how much?
	Yes, No
7.	Did you consume any caffeinated drink in the last 6 h? If Yes, how much?
	Yes, No
8.	How many people are in close proximity (< 3 m) to you RIGHT NOW?
9.	How bright is your immediate environment RIGHT NOW?
	**Too bright, Sufficiently bright, A bit dark, Very dark**
10.	How noisy is the environment you are in RIGHT NOW?
	**Very quiet, Quiet, Noisy, Very noisy.**
**The following questions were taken from the reduced version of the Morningness/Evenningess questionnaire** [89].	
11.	Approximately what time would you get up if you were entirely free to plan your day?
	**5:00–6:30, 6:30–7:45, 7:45–9:45, 9:45–11:00, 11:00-12:00**
12.	On a regular day, during the first half hour after you wake up in the morning, how do you feel?
	**Very tired, Fairly tired, Fairly refreshed, Very refreshed**
13.	On a regular day, at approximately what time in the evening do you feel tired, and, as a result, in need of sleep?
	**20:00–21:00, 20:00–22:15, 22:15-12:45, 12:45–2:00, 2:00–3:00**
14.	On a regular day, at approximately what time of day do you usually feel your best?
	**5:00–8:00, 8:00–10:00, 10:00–17:00, 17:00–22:00, 22:00–5:00**
15.	One hears about ‘morning types’ and ‘evening types’. Which one of these types do you consider yourself to be?
	**Definitely an evening type, Rather more an evening type than a morning type, Rather more a morning type than an evening type, Definitely a morning type**

## Appendix E. Justifications for using GLMM for false recall (as opposed to *t*-test/ANOVA)

The number of critical lures falsely recalled by a participant is count data, ranging from 0 to anything from 8 to 20 (depending on how many DRM lists were shown). In Payne *et al*. [33], for instance, the mean number of lure recalls was approximately 3.25 (out of 8). Count data with a low mean almost never approximates a normal distribution, because it is truncated at 0 (i.e. negative scores are impossible) and is skewed to the right [139]. Parametric tests like *t*-test and ANOVA assume a normal distribution, so they are unlikely to be suitable for false recall data. GLMM, on the other hand, does not assume normal distribution [140]. In addition, GLMM has numerous advantages over a *t*-test or an ANOVA; for instance, it can take by-participant and by-item variance into account, giving researchers the ability to test whether the effect of an independent variable generalizes across participants and items (e.g. [141]).

## Appendix F. Outputs from the exploratory generalized mixed-effect model examining the effects of intrusions, correct recall per list, Interval (Immediate versus Delay), and Test Time (AM versus PM) in false recall

**Table:**

fixed effects	*estimate*	*SE*	*z*	*p*-value
intercept	−3.543	0.09	−38.60	<0.001
intrusions	0.034	0.01	6.01	<0.001
correct recall/list	0.799	0.03	31.85	<0.001
Test Time	0.092	0.07	1.32	0.187
Interval	0.039	0.07	0.55	0.582
correct recall/list×Test Time	−0.062	0.02	−2.78	0.005
correct recall/list×Interval	−0.127	0.02	−5.70	<0.001
Interval×Test Time	−0.095	0.07	−1.37	0.170
correct recall/list×Interval×Test Time	0.062	0.02	2.80	0.005
