# Correction to: ‘A registered report testing the effect of sleep on Deese–Roediger–McDermott false memory: greater lure and veridical recall but fewer intrusions after sleep’ (2023) by Mak *et al.*

**DOI:** 10.1098/rsos.261477

**Published:** 2026-11-11

**Authors:** Matthew H. C. Mak, Alice O'Hagan, Aidan Horner, Gareth Gaskell

**Affiliations:** ^1^ University of Warwick, Coventry, UK; ^2^ University of Oxford, Oxford, UK; ^3^ University of York, York, UK

**Keywords:** sleep, false memory, Deese-Roediger-McDermott, recall, gist abstraction, spreading activation

*R. Soc. Open Sci.* **10**, 220595 (Published online 6 December 2023). (https://doi.org/10.1098/rsos.220595)

In early 2026, a reader brought to the attention of the lead author an error in the specification of the confirmatory mixed-effects models in the published registered report by Mak *et al.* [1].

## 1. Description of the issue

In our recall dataset, the number of intrusions differed significantly as a function of test time, such that participants tested in the evening produced significantly more intrusions than those tested in the morning. As pre-registered, the number of intrusions was included as a covariate in the key analyses of false and veridical memory. However, the Stage 1 report did not fully specify the random-effects structure to be used when including this covariate.

When conducting the analyses for the Stage 2 report, the lead author, who was responsible for implementing the models, incorrectly assumed that, because the number of intrusions varied between participants, it was appropriate to include a by-participant random slope for this predictor in the confirmatory models. However, as noted by the reader and confirmed by the lead author subsequently, this variable is constant within each participant (i.e. each participant only has a single observed value for intrusions) and therefore does not vary at the within-subject level. Consequently, including a random slope for this predictor is not statistically appropriate, as it is not distinguishable from the random intercept and may yield unstable estimates.

## 2. Reanalysis

We re-ran the critical models for both false and veridical memory by removing the inappropriate by-participant random slope for the number of intrusions. The revised models retain the same fixed-effects structure but include only appropriate random-effects terms. Table 1 presents the model summaries for both the original and updated models for the confirmatory analysis on the false recall of critical lures.

**Table 1.** Model summaries for the original and updated models on false memory.

	original model (Table 4 in Mak et al.)				updated model			
model specification	intrusions + interval*test.time + (1 | lure) + (intrusions | participant ID)				intrusions + interval*test.time + (1 | lure) + (1 | participant ID)			
predictor	*β*	SE	*z*	*p*	*β*	SE	*z*	*p*
intercept	−2.328	0.110	−21.253	<0.001*	−2.289	0.108	−21.262	<0.001*
intrusions	0.034	0.006	5.487	<0.001*	0.025	0.004	5.856	<0.001*
interval (immediate vs. delay)	0.039	0.042	0.943	0.346	0.037	0.043	0.856	0.392
test time (AM vs. PM)	0.035	0.041	0.850	0.395	0.021	0.042	0.495	0.620
interval × test time	−0.084	0.041	−2.046	0.041*	−0.078	0.042	−1.852	0.064
pairwise comparisons								
AM vs. PM	−0.098	0.114,	−0.859	0.390	−0.114	0.118	−0.968	0.331
sleep vs. wake	0.239	0.119	2.00	0.045	0.198	0.121	1.637	0.102

In the updated model, the key interval × test time interaction no longer remains statistically significant (*p* = 0.064). We then followed our registered report by conducting pairwise comparisons between (1) the immediate-AM and immediate-PM groups and (2) sleep and wake groups. As shown in the bottom rows of table 1, the pairwise comparison between the sleep and wake groups no longer remains statistically significant in the updated model.

Turning to the recall of studied words, table 2 presents the model summaries for both the original and updated models for the veridical memory analysis.

**Table 2.** Model summaries for the original and updated models on veridical memory.

	original model (Table 4 in Mak et al.)				updated model			
model specification	intrusions + interval*test.time + (1 | lure) + (intrusions | participant ID)				intrusions + interval*test.time + (1 |lure) + (1 | participant ID)			
predictors	*β*	SE	*z*	*p*	*β*	SE	*z*	*p*
intercept	−2.373	0.075	−31.705	<0.001*	−2.40	0.072	−33.548	<0.001*
intrusions	−0.007	0.005	−1.372	0.170	−0.001	0.004	−0.231	0.817
interval (immediate vs delay)	0.307	0.040	7.721	<0.001*	0.319	0.041	7.837	<0.001*
test time (AM vs. PM)	0.026	0.037	0.691	0.489	0.023	0.038	0.601	0.548
interval × test time	−0.124	0.037	−3.324	<0.001*	−0.116	0.038	−3.030	0.002*
pairwise comparisons								
AM vs. PM	−0.196	0.104	−1.881	0.060	−0.186	0.106	−1.758	0.078
sleep vs. wake	0.299	0.107	2.797	0.005	0.278	0.111	2.500	0.013

In the updated model, the critical interval × test time interaction remains statistically significant (*p* = 0.002), as does the corresponding pairwise comparison between sleep and wake conditions (*p* = 0.013), indicating that the sleep group outperformed the wake group in veridical recall, above and beyond the difference between the immediate-AM and immediate-PM groups.

## 3. Impact on conclusions

For false recall, although the reanalysis showed that the direction of the interval × test time interaction remains consistent with that reported in the original registered report and the evidential shift in the reanalysis is small, the effect no longer reaches conventional levels of statistical significance in the updated model. In addition, the corresponding pairwise comparison between the sleep and wake groups at the delayed timepoint is also no longer statistically significant. Taken together, these updates suggest that the effect of sleep (relative to wake) on false recall is less robust than originally reported in our article, and is consistent with recent evidence reporting a negligible effect of sleep on Deese–Roediger–McDermott false recall [2]. As such, any conclusions regarding a reliable influence of sleep on false recall are not warranted based on the corrected analyses.

The pattern of results for veridical memory remains unchanged, with the critical interval × test time interaction remaining statistically significant in the updated model. This preserves the original conclusion that sleep-related consolidation processes benefit veridical memory retention.

Overall, the correction refines the interpretation of the findings by narrowing the scope of the conclusions: while evidence for sleep-related effects on veridical memory remains robust, evidence for a comparable effect on false recall is weak (see also [2]).

## Data Availability

The corrected model specification and updated analysis script have been made available at OSF [3].
